# Characterizing spatial immune architecture in metastatic melanoma using high-dimensional multiplex imaging

**DOI:** 10.3389/fimmu.2025.1560778

**Published:** 2025-04-29

**Authors:** Joel Eliason, Santhoshi Krishnan, Yasunari Fukuda, Matias A. Bustos, Dan Winkowski, Sungnam Cho, Akshay Basi, Regan Baird, Elizabeth A. Grimm, Michael A. Davies, Dave S. B. Hoon, Arvind Rao, Jared K. Burks, Suhendan Ekmekcioglu

**Affiliations:** ^1^ Department of Computational Medicine and Bioinformatics, University of Michigan, Ann Arbor, MI, United States; ^2^ Kindai University, Nara Hospital, Nara, Japan; ^3^ Saint John’s Health Center, Santa Monica, CA, United States; ^4^ Visiopharm A/S, Horsholm, Denmark, Denmark; ^5^ Department of Melanoma Medical Oncology, Melanoma Medical Oncology, MD Anderson Cancer Center, University of Texas, Houston, TX, United States; ^6^ Department of Leukemia, MD Anderson Cancer Center, Houston, TX, United States; ^7^ Department of Biostatistics, University of Michigan, Ann Arbor, MI, United States; ^8^ Department of Biomedical Engineering, University of Michigan, Ann Arbor, MI, United States; ^9^ Department of Radiation Oncology, University of Michigan, Ann Arbor, MI, United States

**Keywords:** tumor immune microenvironment (TIME), immune exclusion, spatial immune profiling, inflammatory biomarkers, melanoma progression, prognostic immune signatures, inflammatory signaling pathways, immune cell crosstalk

## Abstract

**Introduction:**

Immune checkpoint inhibitors (ICIs) have significantly improved survival for patients with metastatic melanoma, yet many experienceresistance due to immunosuppressive mechanisms within the tumor immune microenvironment (TIME). Understanding how the spatial architecture of immune and inflammatory components changes across disease stages may reveal novel prognostic biomarkers and therapeutic targets.

**Methods:**

We performed high-dimensional spatial profiling of two melanoma tissue microarrays (TMAs), representing Stage III (*n* = 157) and Stage IV (*n* = 248) metastatic tumors. Using imaging mass cytometry (IMC) and multiplex immunofluorescence (mIF), we characterized the phenotypic, functional, and spatial properties of the TIME. Cellular neighborhoods were defined by inflammatory marker expression, and spatial interactions between immune and tumor cells were quantified using nearest-neighbor functions (G-cross). Associations with survival were assessed using Cox proportional hazards models with robust variance estimation.

**Results:**

Stage IV tumors exhibited a distinct immune landscape, with increased CD74- and MIF-enriched inflammatory neighborhoods and reduced iNOS-associated regions compared to Stage III. Cytotoxic T lymphocytes (CTLs) and tumor cells were more prevalent in Stage IV TIME, while B cells and NK cells were depleted. Spatial analysis revealed that CTL–Th cell, NK–T cell, and B–NK cell interactions were linked to improved survival, whereas macrophage aggregation and excessive B–Th cell clustering in inflammatory regions correlated with worse outcomes. Organ-specific analyses showed that CTL infiltration near tumor cells predicted survival in gastrointestinal metastases, while NK–T cell interactions were prognostic in lymph node and skin metastases.

**Discussion:**

Our results reveal stage-specific shifts in immune composition and spatial organization within the melanoma TIME. In advanced disease, immunosuppressive neighborhoods emerge alongside changes in immune cell localization, with spatial patterns of immune coordination—particularly involving CTLs, NK cells, and B cells—strongly predicting survival. These findings highlight spatial biomarkers that may refine patient stratification and guide combination immunotherapy strategies targeting the inflammatory architecture of the TIME.

## 1 Introduction

Invasive melanoma is the least common but deadliest form of skin cancer. It is estimated that 200,340 new cases of melanoma will be diagnosed in 2024 (1). In the United States, melanoma is the fifth most common cancer in both men and women across all age groups and the third most common cancer among individuals aged 20–39 (2). Over the past decade, there has been a significant increase in the overall five-year survival rate for patients with metastatic melanoma. Between 2014 and 2018, the melanoma mortality rate declined by approximately 7% per year in adults younger than 50 and by about 5% per year in older adults (3). This improvement is largely attributed to earlier diagnosis, advancements in surgical techniques, and the development of novel therapeutic approaches. In particular, molecularly targeted therapies, vaccines, adoptive T-cell therapy, and immune checkpoint inhibitors (ICIs) have revolutionized melanoma treatment (4).

Among these, ICIs have had the most significant impact by inducing durable responses and conferring long-term survival benefits through enhanced tumor immunity (5–7). However, despite their efficacy, a subset of patients experiences either primary resistance, where they fail to respond initially, or acquired resistance, where tumors progress after an initial response to ICIs (8). These resistance mechanisms are primarily driven by immune evasion strategies that alter tumor-immune interactions, including T cell exhaustion, regulatory T cell activation, recruitment of myeloid-derived suppressor cells and macrophage polarization toward the immunosuppressive M2 phenotype (9–11). To develop more effective therapeutic strategies that enhance immunotherapy outcomes, it is critical to first gain a comprehensive understanding of both the phenotypic and functional spatial organization of the tumor immune microenvironment (TIME).

The complexity and heterogeneity of the TIME pose significant challenges in identifying molecular biomarkers in metastatic melanoma and understanding how the TIME influences responses to ICI therapy. Tumor cells, as major constituents of the TIME, play a pivotal role in shaping the immune landscape by secreting tumor antigens and modulating immune cell function, often creating an unfavorable microenvironment that hinders immune responses. Previous studies by our group have demonstrated that inflammatory marker expression in metastatic melanoma is associated with prognostic indicators such as recurrence-free survival (RFS) and overall survival (OS) (12–16).

The present study aims to provide an extensive analysis of the TIME in advanced melanoma, with a particular focus on the TIME’s cellular components and their spatial organization in relation to inflammatory marker expression within the immediate cellular neighborhood.

## 2 Materials and methods

### 2.1 Patient samples and datasets

Two independent tissue microarrays (TMAs) were utilized, representing non-primary metastatic melanoma lesions: Stage III (regional metastases) and Stage IV (distant metastases). Under IRB-approved protocols, cases with available archival material and clinical follow-up exceeding five years were included. Clinical records were reviewed for key events, including local recurrence, distant metastasis patterns, and overall survival (OS).

- the Stage III TMA, developed at the Saint John’s Cancer Institute (SJCI), was prepared from formalin- fixed paraffin-embedded (FFPE) lymph node tumors. It contained two core samples per patient from 157 Stage III patients, with annotated clinical outcomes and follow-up data.
- the Stage IV TMA, also developed at SJCI, comprised 393 core samples from 248 Stage IV patients. These samples, derived from FFPE melanoma tissues obtained from Saint John’s Health Center (SJHC), represented a range of distant metastatic sites. Clinical outcomes and follow-up data were sourced from the SJCI melanoma database. A hematoxylin and eosin (H&E) slide was prepared for each sample, and all specimens were reviewed by a dedicated dermatopathologist to ensure tumor viability. Tumor regions of interest were mapped to FFPE blocks before TMA construction.

To enable simultaneous staining and data acquisition across the cohort, the Stage IV TMA was constructed using 1 mm cylindrical cores, distributed in duplicate and randomized to minimize spatial bias. After accounting for core loss during sectioning, 393 evaluable samples remained, representing metastatic sites such as lung, gastrointestinal tract, lymph nodes, skin, and other distant locations.

**Figure 1:**
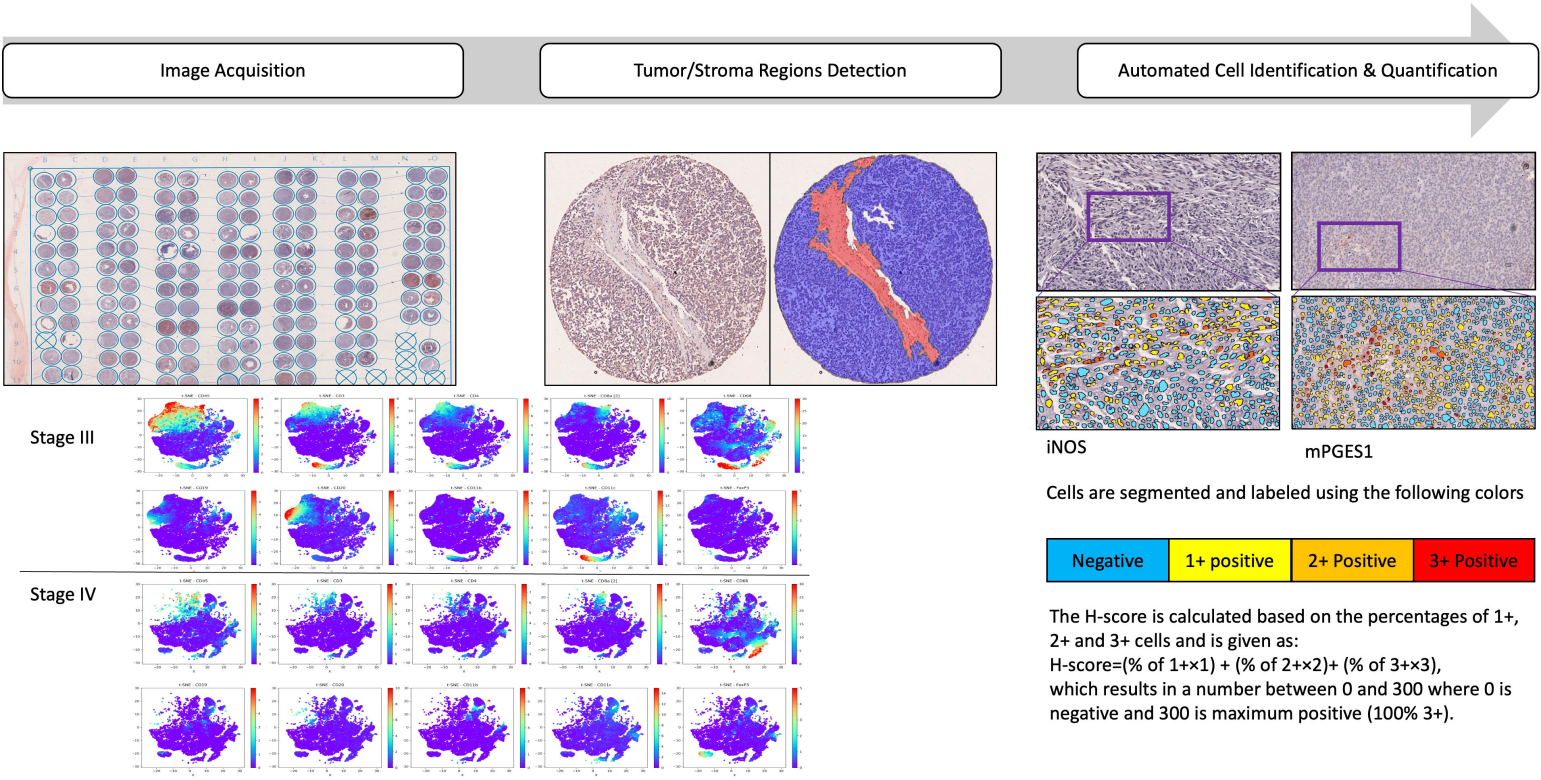
Analysis pipeline to characterize the spatial immune landscape of Stage III and IV melanomas. Schematic of the IMC data acquisition of two consecutive slices of two TMAs containing 2 biopsy cores from a total of 157 patients with stage III and 393 samples from 248 patients with stage IV metastatic melanoma. Samples were stained with a protein panel, segmented for tumor/stroma regions detection, followed by cellular identification and quantifications. t-distributed stochastic neighbor embedding (t-SNE) of data was derived from CyTOF of tumor samples, labeled by cell type and the signal intensities of individual markers.

Staging for both TMAs followed AJCC guidelines at the time of melanoma diagnosis (17). Clinicopathological characteristics are summarized in **Tables 1**, **2**. A diagram of our analysis pipeline is shown in **Figure 1**.

**Table 1:** Summary of key clinical and demographic characteristics for stage III TMA patients.

Stage III Patient Characteristics	N = 157
Age (years)	52 (24)
(Missing)	10
Gender	
Female	61 (41%)
Male	86 (59%)
(Missing)	10
Breslow Thickness (mm)	2.20 (2.65)
(Missing)	50
Has Ulceration	31 (31%)
(Missing)	57
Observation Period (months)	100 (145)
(Missing)	26
Immunotherapy Timing	
No Treatment	32 (22%)
Pre-Surgery (Neoadjuvant)	29 (20%)
Post-Surgery (Adjuvant)	60 (41%)
(Missing)	10
Chemotherapy Timing	
No Treatment	112 (76%)
Pre-Surgery (Neoadjuvant)	10 (6.8%)
Post-Surgery (Adjuvant)	25 (17%)
Both Pre- & Post-Surgery	0(0%)
(Missing)	10
Radiation Timing	
No Treatment	95 (65%)
Pre-Surgery (Neoadjuvant)	9 (6.1%)
Post-Surgery (Adjuvant)	40 (27%)
Both Pre- & Post-Surgery	3 (2.0%)
(Missing)	10

^1^ Median (IQR); n (%).

**Table 2:** Summary of key clinical and demographic characteristics for stage IV TMA patients.

Stage IV Patient Characteristics	N = 248
Age (years)	56 (18)
Gender	
Female	79 (32%)
Male	169 (68%)
Metastatic Site	
GI	42 (17%)
Skin	50 (20%)
Lymph Node	30 (12%)
Lung	39 (16%)
Other Metastatic Site	87 (35%)
Breslow Thickness (mm)	1.90 (1.70)
(Missing)	67
Has Ulceration	60 (39%)
(Missing)	95
Observation Period (months)	56 (80)
(Missing)	44
Lymph Nodes Positive	1.00 (1.00)
(Missing)	48

^1^ Median (IQR); n (%).

### 2.2 Imaging mass cytometry by time-of-flight

Imaging Mass Cytometry (IMC) data were acquired using the Hyperion Imaging System (Fluidigm) coupled to a Helios mass cytometer (Fluidigm). All imaging acquisitions followed the manufacturer’s protocols. Briefly, the TMA tissue slide underwent laser ablation at a resolution of approximately 1 *µ*m and a frequency of 200 Hz. A 1 × 1 mm region of interest (ROI) was selected from each TMA core, and data acquisition was performed in three batches. IMC data were stored as MCD and text files, with each individual MCD file manually reviewed to confirm staining presence across all channels. Poor-quality TMA cores were excluded from further analysis.

CyTOF-based IMC profiling utilized TMA slides containing multiple samples stained with a cocktail of 35 antibodies labeled with unique metal isotopes (**Supplementary Table S2**). A high-energy laser ablated the slides, converting the tissue into ionized trails to generate isotope counts for each ablated spot. These data were then reconstructed into image stacks, capturing the staining patterns for each antibody. The antibody panel for melanoma TIME characterization was designed based on prior studies and commonly used clones in immunohistochemistry. The selected antibodies targeted phenotypic markers to define immune infiltrates (e.g., CD3, CD4, CD8, CD20, and CD68), melanoma markers (SOX10 and S100), structural markers (e.g., CD31), and functional markers (e.g., GzmB, Ki67). Additionally, antibodies against key inflammatory pathways, including CD74, MIF, iNOS, and mPGES1, were incorporated. To ensure specificity and affinity, the panel was validated in spleen, thymus, and melanoma tissue samples.

Following tumor and stromal segmentation, individual cells were identified and classified based on staining intensity, quantified using the H-score:


$$\text{H}-\text{score }=\;\left(\%\text{ of }\;1^{+}\;\times 1\right)\;+\;\left(\%\text{ of}\;2^{+}\;\times 2\right)\;+\;\left(\%\text{ of }3^{+}\;\times 3\right)$$

The H-score ranges from 0 to 300, where 0 indicates no staining (negative) and 300 represents maximum staining (100% of cells stained at 3^+^ intensity).

**Figure 2:**
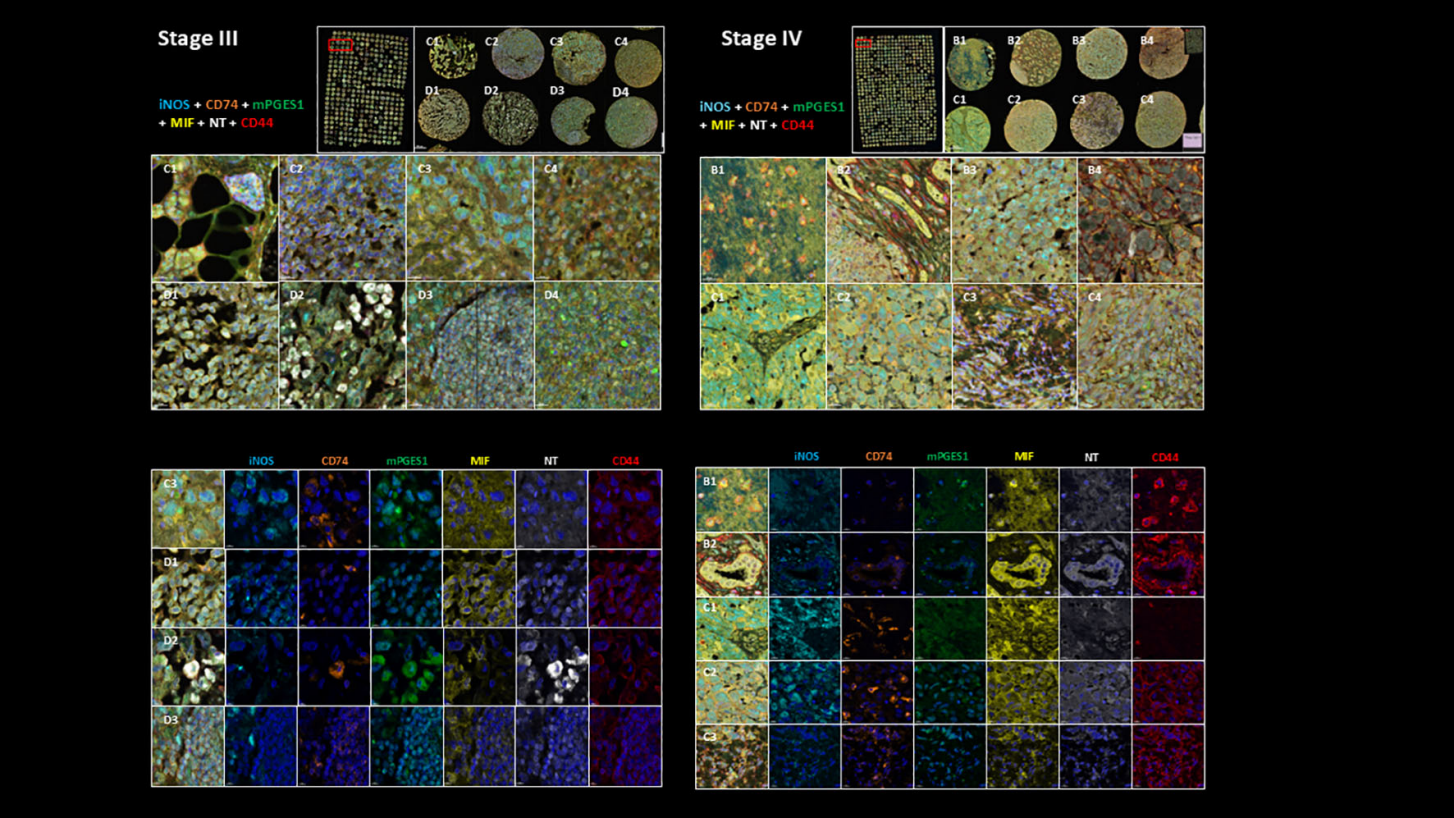
Representative multiplex immunostaining of Stage III and Stage IV melanoma TMA cores showing inflammatory markers (iNOS, mPGES1, MIF, NT, CD44, and CD74; scale bars=20 *µ*m). Top part of the figure shows their expression in sample cores combined and bottom part shows their individual expression characteristics.

The heterogeneity and distribution of various cell types are determined and visualized using t-distributed stochastic neighbor embedding (t-SNE) across Stages III and IV. This enables visualization of high- dimensional data in 2 or 3 dimensions, while preserving the innate structure and variance of the data. For the study, t-SNE plots were generated using CyTOF-derived tumor data, with cells labeled by type and individual marker signal intensities (**Figure 2**).

### 2.3 Multiplex immunofluorescence staining and image acquisition

Multiplex immunofluorescence (mIF) staining was performed using the Akoya Biosciences Opal 7-Color Manual IHC Kit (catalog number NEL811001KT) following the manufacturer’s specifications. Briefly, slides were deparaffinized, rehydrated, and subjected to melanin bleaching according to the protocol described by (18). Antigen retrieval was carried out using the EZ-Retriever V.3 system (BioGenex, Fremont, CA, USA) with AR6 buffer (Akoya Biosciences) at 95°C for 15 minutes between antibody staining cycles, followed by a 15-minute cooling period at room temperature.

The antibody panel and corresponding fluorophores were applied at the following concentrations: CD74 (clone PIN.1, 1:100; Opal 620), CD44 (clone 156-3C11, 1:100; Opal 690), polyclonal MIF (1:100; Opal 570), mPGES1 (1:100; Opal 520), iNOS (1:100; Opal 480), and NT (1:100; Opal 780). DAPI was used for nuclear staining, and slides were mounted with Shandon Immu-Mount (Thermo Fisher Scientific, catalog number 28-600-42).

Image acquisition was performed using the Akoya Biosciences Vectra Polaris automated quantitative pathology imaging system. Cell segmentation and downstream phenotyping analysis were conducted using Visiopharm. A representative example of multiplex immunostaining of Stage III and Stage IV melanoma TMA cores, showing inflammatory markers (iNOS, mPGES1, MIF, NT, CD44, and CD74), is displayed in **Figure 2**.

### 2.4 Multiplex imaging alignment and cellular neighborhood analysis

mIF and IMC images were aligned using the TissueAlign co-registration workflow in Visiopharm (19). Following alignment, cellular neighborhoods were defined using a structured workflow.

First, in each TMA sample, AI-driven nuclear segmentation in Visiopharm identified individual nuclei based on DAPI signal in the mIF image. To approximate cellular boundaries, each nuclear object was expanded by 3 microns. Cells were then classified as positive for specific biomarkers or biomarker combinations using intensity thresholding. Once specific phenotypic cell populations were identified, their surrounding neighborhoods (20–80 microns) were defined as Regions of Interest (ROIs). These neighborhood ROIs were then transferred to the co-registered IMC image for further analysis.

For IMC-based cellular characterization, an AI-driven algorithm was applied to the DNA channel (iridium signal) to detect nuclei. Similar to mIF processing, nuclear boundaries were expanded by 3 microns to approximate cellular objects. Cells were then classified based on marker expression, with phenotype assignments determined through intensity thresholding. To ensure accuracy, phenotypic classifications were validated via visual inspection, and manual adjustments were made as needed.

### 2.5 Statistical analysis

To compare the proportions of cell types and inflammatory neighborhoods between Stage III and Stage IV melanoma, we used beta regression models with false discovery rate (FDR) correction for multiple comparisons. Beta regression was chosen as it appropriately models proportion data constrained between 0 and 1. Groups with zero variance were excluded from analysis to ensure valid model estimation. For each comparison, the beta regression model included stage as the independent variable and the proportion of the respective feature as the dependent variable. Beta regression models were fit using the betareg R package (20). P-values were adjusted using the Benjamini-Hochberg method (21), and statistical significance was defined as *p <* 0.05. Boxplots and heatmaps were used to visualize the distributions of proportions, with significance markers displayed where appropriate. Cox proportional hazards models were fitted using the R package survival (22), and Kaplan-Meier plots were generated using the R package survminer (23). To account for intra-patient correlation when patients contributed more than one sample, as in the Stage IV TMA, Cox regression models were fitted with robust standard errors using the cluster argument in the coxph function. This approach adjusts variance estimates to account for the non-independence of observations within the same patient. Other plots were created using the tidyverse packages in R (24). All statistical analyses performed on the Stage IV TMA were performed on samples from all organs pooled together, unless indicated otherwise (such as in Section 3.9). All samples from the Stage III and Stage IV TMA were used for all statistical analyses.

#### 2.5.1 Spatial analysis

For the analysis of spatial dispersion and clustering between different pairs of cell types, we estimated the G-cross nearest neighbor distance function *G_i,j_* (*r*), which is a function of the distance *r*, with *i* and *j* indicating the two types of cells (25, 26). Briefly, *G_i,j_* (*r*) represents the cumulative distribution function of the distance from a typical point of type *i* to the nearest point of type *j*. Mathematically, this can be expressed as:


$$G_{i,j}(r)=1-\text{exp}(-\alpha_{j}\pi r^{2})$$

where the subscripts *i* and *j* indicate that the spatial distribution of cell type *j* relative to cell type *i* is being computed, *r* refers to the distance from the reference cell type, and *α_j_* is the overall density of cell type *j* on the slide.

We estimated *G_i,j_* (*r*) using the Gcross function in the R package spatstat (27). To assess the clinical significance of the estimated *G_i,j_* (*r*) across Stages III and IV, we extracted the *G_i,j_* (*r*) estimates at various radii for each cell type pair and used them as independent variables in a univariate Cox proportional hazards (CoxPH) model. For the Stage IV organ-wise metastases survival analyses, we used the area under the curve (AUC) of the estimated *G_i,j_* (*r*) values at various radii as the independent variable in our CoxPH model. These models were fitted using the coxph function in the R package survival (22).

## 3 Results

### 3.1 Summary of stage III patient characteristics

The Stage III TMA cohort included 157 patients with a median age of 52 years (IQR: 24 years) (**Table 1**). The cohort was 59% male and 41% female. Median Breslow thickness was 2.20 mm (IQR: 2.65 mm), though data were missing for a substantial number of patients (n = 50). Ulceration was reported in 31% of cases, while 57 patients had unknown ulceration status. The median observation period was 100 months (IQR: 145 months). Most patients (76%) did not receive chemotherapy, while 35% received radiation therapy. Immunotherapy use was variable, with 41% receiving adjuvant treatment, 20% receiving neoadjuvant treatment, and 18% receiving both.

The median overall survival for Stage III TMA patients was 20.2 months (95% CI: 15.3–61.7) (**Supplementary Table S3**). The estimated 5-year survival probability was 41.5%, while the 10-year survival probability was 27.9%.

### 3.2 Clinical and demographic characteristics of stage IV TMA patients

The Stage IV TMA cohort included 248 patients with a median age of 56 years (IQR: 18 years) (**Table 2**). The cohort was 68% male and 32% female. Metastatic sites varied, with 35% of patients having metastases at other locations, followed by skin (20%), gastrointestinal (GI) tract (17%), lung (16%), and lymph nodes (12%). Median Breslow thickness was 1.90 mm (IQR: 1.70 mm), though data were missing for 67 patients. Ulceration was present in 39% of cases, with ulceration status unknown for 95 patients. The median observation period was 56 months (IQR: 80 months), though 44 patients had missing data. The median number of positive lymph nodes was 1.00 (IQR: 1.00), with lymph node status unavailable for 48 patients.

**Figure 3:**
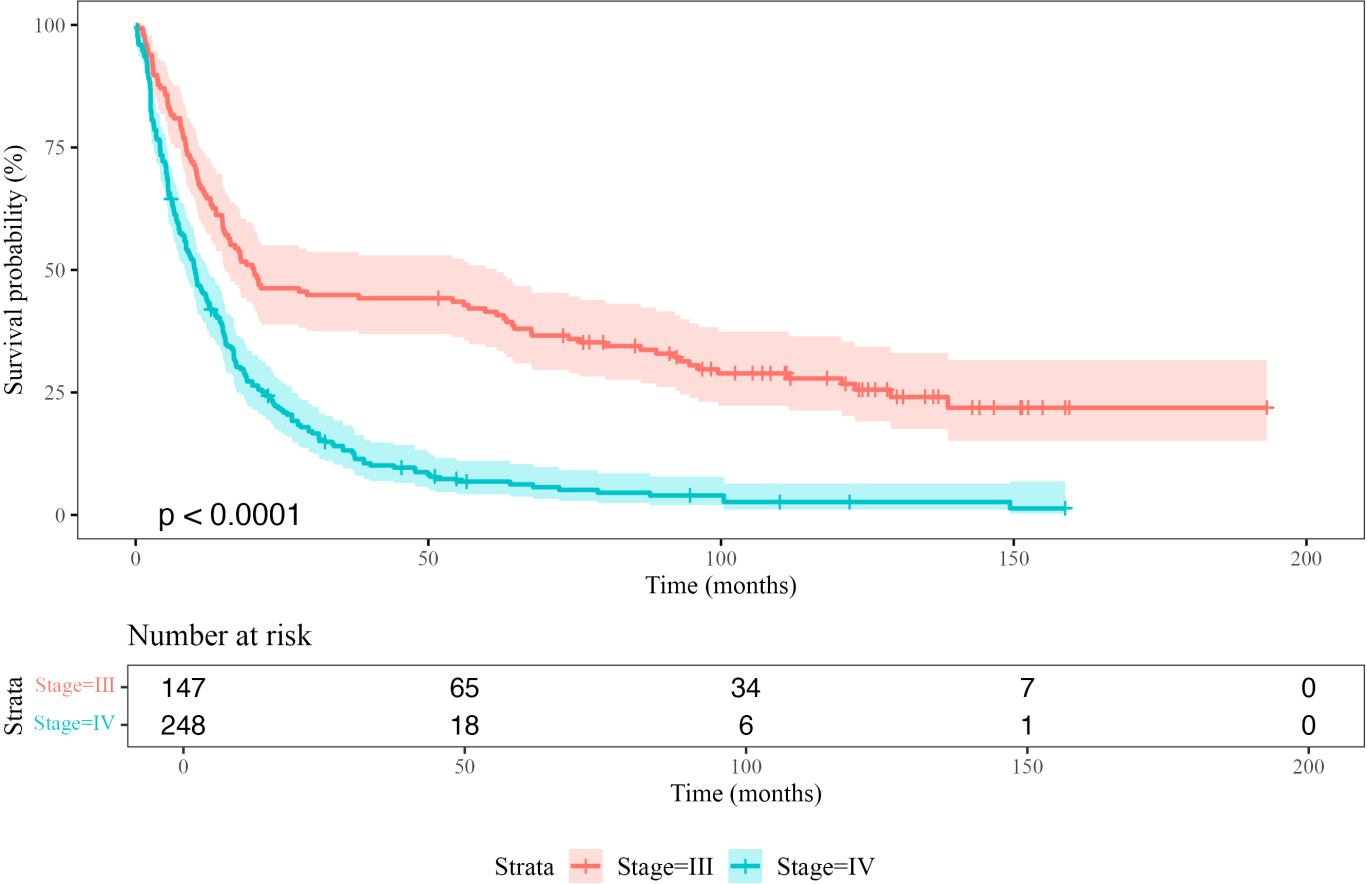
Kaplan-Meier survival curves comparing overall survival (OS) probability between Stage III and Stage IV patients. The shaded regions represent 95% confidence intervals. The p-value indicates the statistical significance of the difference between the survival distributions (log-rank test). The risk table below the plot shows the number of patients at risk at each time point.

The median overall survival (OS) for Stage IV melanoma patients was 10.2 months (95% CI: 8.6–12.4), with a 5-year survival probability of 6.8% and a 10-year survival probability of 2.6% (**Supplementary Table S4**). Median disease-free survival (DFS) was 18.7 months (95% CI: 15.0–29.2). The 5-year DFS probability was 31.8%, decreasing to 20.2% at 10 years (**Supplementary Table S5**). Kaplan-Meier curves showing the empirical overall survival probabilities for patients from both the Stage III and IV TMA are shown in **Figure 3**.

### 3.3 Differences in immune cell composition between stages

**Figure 4:**
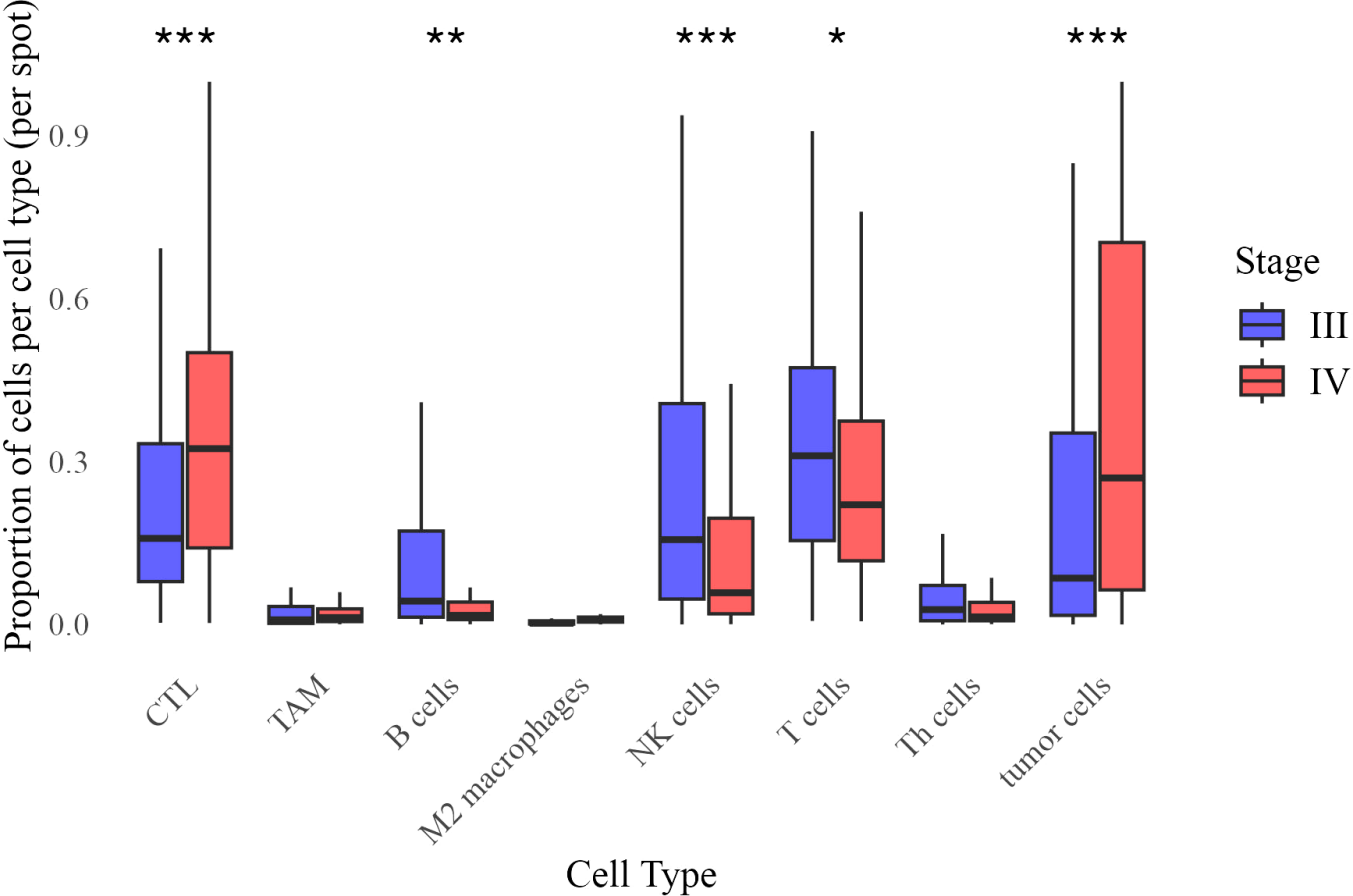
Distribution of cell type proportions across Stage III and Stage IV melanoma. Each boxplot represents the proportion of a given cell type per image, grouped by stage. Statistical significance of difference in mean proportion was assessed using a beta regression model, with significance markers (*,**, ***) indicating significant differences between stages after false discovery rate correction. Significance levels are denoted as follows: ∗ ∗ ∗ (*p <* 0.001), ∗ ∗ (*p <* 0.01), and ∗ (*p <* 0.05).

To characterize changes in the tumor immune microenvironment between Stage III and IV melanoma, we quantified the relative proportions of immune and tumor cells in tumor core biopsies. Beta regression analysis identified significant shifts in cell type distributions (**Figure 4**), where the beta coefficient (*β*) represents the change in log-odds of a cell type’s proportion in Stage IV relative to Stage III.

Stage IV tumors exhibited significantly higher proportions of cytotoxic T lymphocytes (CTL, *β* = 0.474, *p <* 0.001) and tumor cells (*β* = 0.487, *p <* 0.001), while B cells (*β* = −0.365, *p* = 0.007), NK cells (*β* = −0.541, *p <* 0.0001), and overall T cell populations (*β* = −0.188, *p* = 0.044) were reduced. Differences in tumor-associated macrophages (TAM, *p* = 0.109), M2 macrophages (*p* = 0.192), and T helper (Th) cells (*p* = 0.153) were not statistically significant. These results indicate an immune shift in Stage IV, characterized by increased CTL infiltration and decreased adaptive immune populations.

### 3.4 Shifts in inflammatory neighborhoods

**Figure 5:**
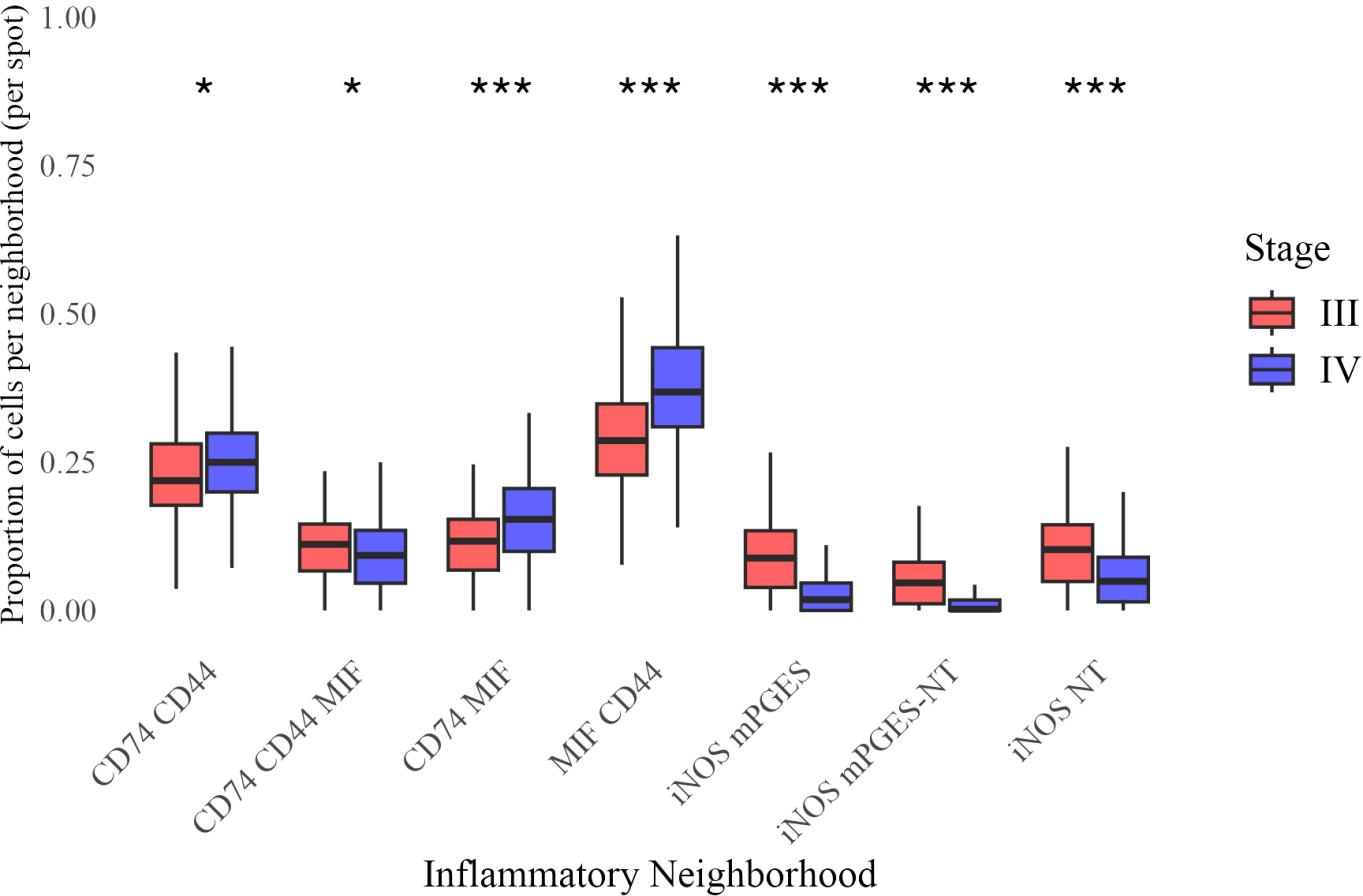
Distribution of neighborhood proportions across Stage III and Stage IV melanoma. Each boxplot represents the proportion of a given inflammatory neighborhood per image, grouped by stage. Statistical significance of difference in mean proportion was assessed using a beta regression model, with significance markers (*, **, ***) indicating significant differences between stages after false discovery rate correction. Significance levels are denoted as follows: ∗ ∗ ∗ (*p <* 0.001), ∗ ∗ (*p <* 0.01), and ∗ (*p <* 0.05).

To examine immune spatial organization, we analyzed seven inflammatory neighborhoods based on prior studies (14, 15, 28–31). Beta regression analysis identified significant differences in their prevalence between Stage III and IV melanoma (**Figure 5**).

Stage IV tumors had a higher prevalence of CD74 CD44 (*β* = 0.0841, *p* = 0.015), CD74 MIF (*β* = 0.306, *p <* 0.0001), and MIF CD44 neighborhoods (*β* = 0.320, *p <* 0.0001), while iNOS mPGES (*β* = −0.772, *p <* 0.0001), iNOS mPGES-NT (*β* = −0.773, *p <* 0.0001), and iNOS NT neighborhoods (*β* = −0.455, *p <* 0.0001) were significantly less frequent. A modest but significant reduction was also observed for CD74 CD44 MIF (*β* = −0.121, *p* = 0.015). These findings indicate a shift toward CD74- and MIF-enriched neighborhoods in Stage IV, accompanied by a loss of iNOS-associated regions.

### 3.5 Immune cell distribution within inflammatory neighborhoods

**Figure 6:**
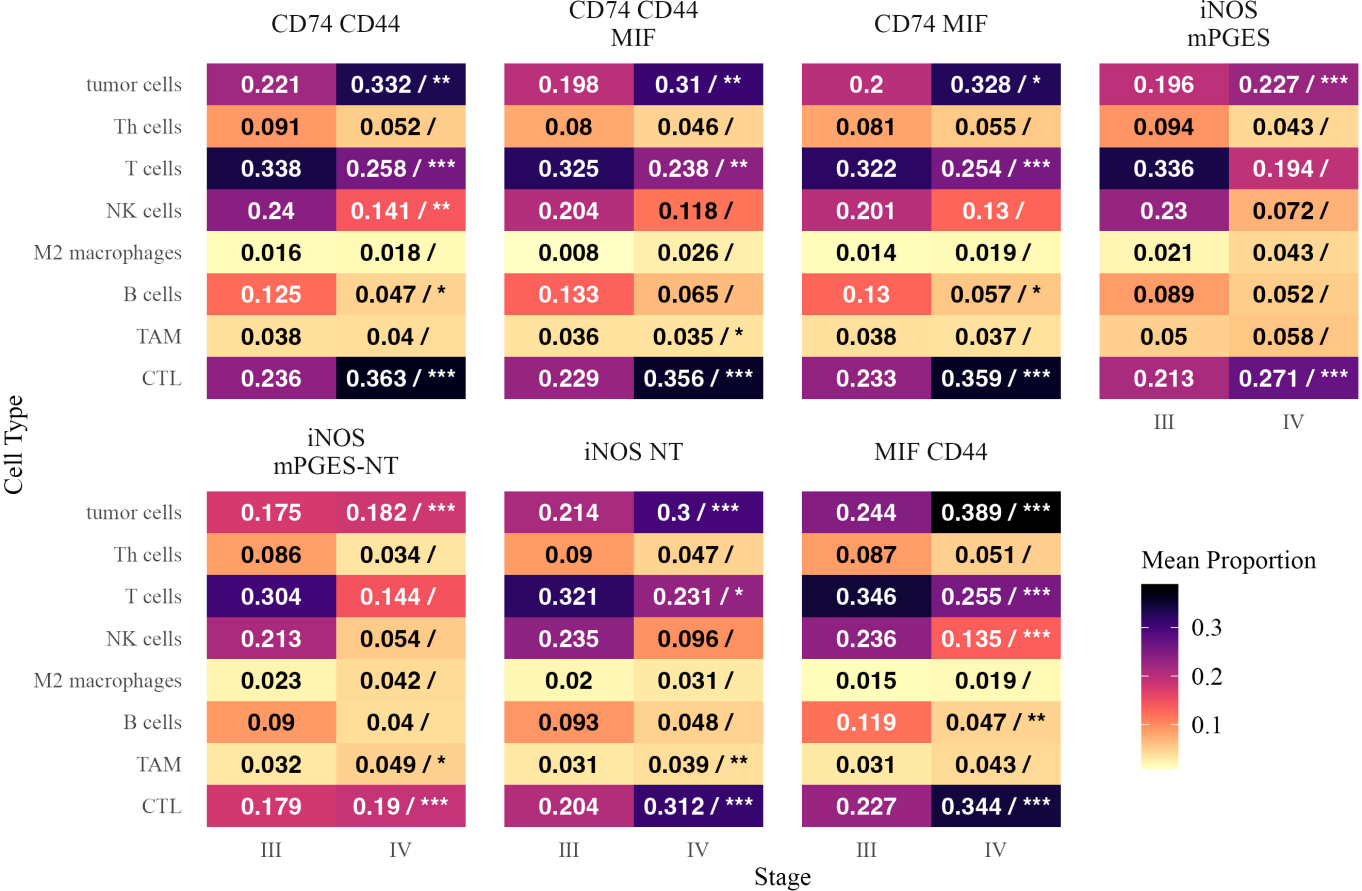
Mean proportions of different cell types within inflammatory neighborhoods in Stage III and Stage IV melanoma. Each tile is annotated with the mean proportion of the corresponding cell type among cells in that neighborhood. In the Stage IV column, a forward slash (“/”) separates the mean proportion from statistical significance markers (*, **, ***), indicating significant differences in mean proportion between stages based on a beta regression model with false discovery rate correction. Significance levels are denoted as follows: ∗ ∗ ∗ (*p <* 0.001), ∗ ∗ (*p <* 0.01), and ∗ (*p <* 0.05). Text color is adjusted for readability.

We next examined the distribution of immune cells as a fraction of all cells in each inflammatory neighborhood in Stage III and IV melanoma (**Figure 6**). CTLs and tumor cells were significantly more abundant in all neighborhoods in Stage IV, suggesting a broader restructuring of the tumor microenvironment.

B cells and NK cells were generally reduced in Stage IV, particularly as a proportion of CD74- and MIF-enriched neighborhoods. B cells were significantly lower in CD74 CD44 (*β* = −0.393, *p* = 0.014) and CD74 MIF (*β* = −0.426, *p* = 0.012), while NK cells were depleted in CD74 CD44 (*β* = −0.367, *p* = 0.0018) and MIF CD44 (*β* = −0.463, *p <* 0.0001). T cells were also significantly reduced as a fraction of multiple neighborhoods, including CD74 MIF (*β* = −0.292, *p <* 0.0001) and MIF CD44 (*β* = −0.359, *p <* 0.0001), despite the overall increase in CTLs. M2 macrophages and Th cells did not show significant enrichment or depletion in any neighborhood.

### 3.6 Neighborhood representation within immune cell populations

**Figure 7:**
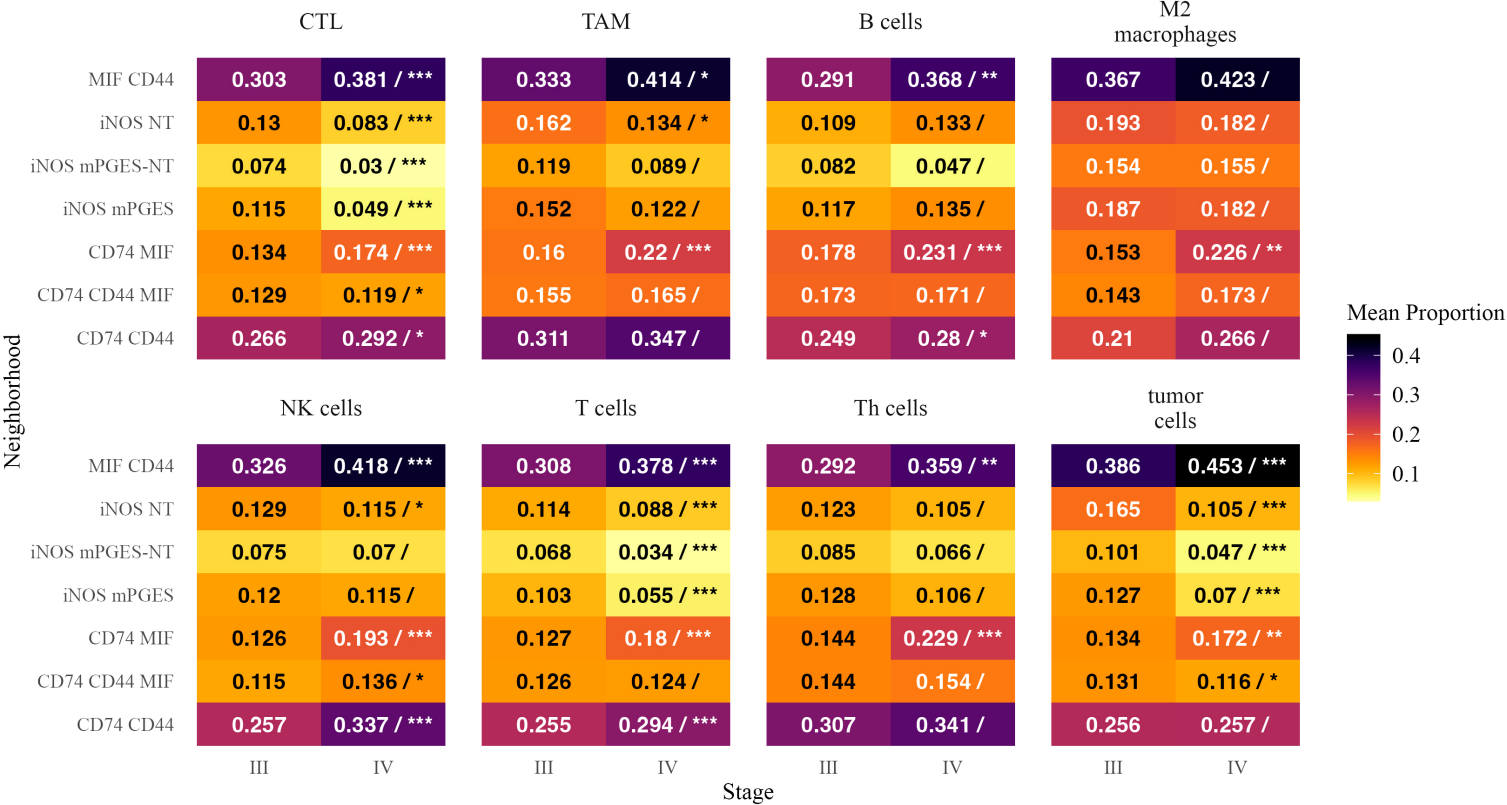
Mean proportions of inflammatory neighborhoods as fractions of cells within each cell type in Stage III and Stage IV melanoma. Each tile is annotated with the mean proportion of the corresponding neighborhood among cells within that cell type. In the Stage IV column, a forward slash (“/”) separates the mean proportion from statistical significance markers (*, **, ***), indicating significant differences in mean proportion between stages based on a beta regression model with false discovery rate correction. Significance levels are denoted as follows: ∗ ∗ ∗ (*p <* 0.001), ∗ ∗ (*p <* 0.01), and ∗ (*p <* 0.05). Text color is adjusted for readability.

To assess how immune and tumor cells partition across neighborhoods, we analyzed the fraction of each cell type residing in different inflammatory neighborhoods (**Figure 7**). A positive estimate indicates a greater fraction of the overall population of a given cell type was located within a specific neighborhood in Stage IV.

CTLs were more frequently found in CD74 MIF (*β* = 0.286, *p <* 0.0001) and MIF CD44 (*β* = 0.241, *p <* 0.0001), suggesting an increased presence of cytotoxic immune responses in these neighborhoods. B cells were more localized within CD74 MIF (*β* = 0.249, *p <* 0.0001) and MIF CD44 (*β* = 0.231, *p* = 0.0016). Tumor cells followed a similar trend, increasing in CD74 MIF (*β* = 0.219, *p* = 0.005) and MIF CD44 (*β* = 0.190, *p <* 0.0001), further supporting their role as hubs of immune activation and tumor presence in advanced disease (32).

Conversely, iNOS- and mPGES-enriched neighborhoods accounted for a smaller fraction of CTLs and T cells in Stage IV. The proportion of CTLs was significantly lower in iNOS mPGES (*β* = −0.804, *p <* 0.0001) and iNOS mPGES-NT (*β* = −0.750, *p <* 0.0001). T cells showed a similar reduction in iNOS mPGES (*β* = −0.684, *p <* 0.0001) and iNOS mPGES-NT (*β* = −0.642, *p <* 0.0001), suggesting a diminished adaptive immune presence in these regions.

MIF CD44 neighborhoods encompassed a greater fraction of NK cells in Stage IV (*β* = 0.251, *p <* 0.0001), while iNOS NT neighborhoods accounted for a smaller proportion of NK cells (*β* = −0.266, *p* = 0.016). Th cells were more frequently found in MIF CD44 (*β* = 0.234, *p* = 0.0035) and CD74 MIF (*β* = 0.424, *p <* 0.0001). M2 macrophages in CD74 MIF made up a larger proportion of their overall cell populations in Stage IV than in Stage III (*β* = 0.360, *p <* 0.0001; *β* = 0.478, *p* = 0.0037).

These findings suggest that in Stage IV melanoma, immune and tumor cells preferentially localize within CD74- and MIF-enriched neighborhoods, while iNOS-associated neighborhoods see a reduced presence of CTLs, T cells, and tumor cells. This redistribution may reflect altered immune engagement and inflammatory signaling in advanced disease.

### 3.7 Spatial immune interactions and their association with patient survival

#### 3.7.1 Spatial interactions in stage III patients

**Figure 8:**
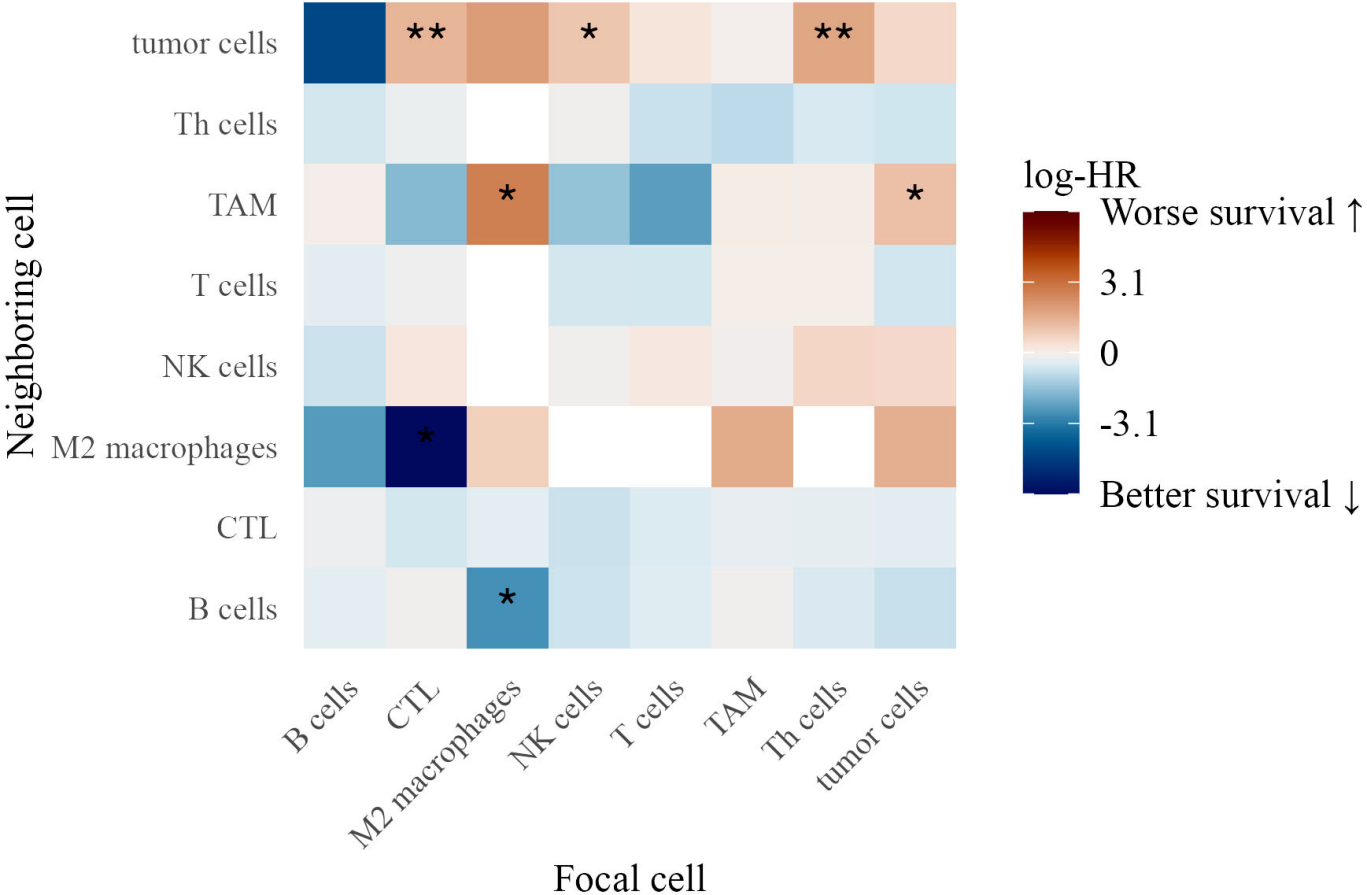
Heatmap of log-hazard ratios estimated using Cox regression, where the G-cross function at 40 *µ*m between each pair of cell types in Stage III patients serves as a predictor of survival time. Significance levels of hazard ratios are denoted as follows: ∗ ∗ ∗ (*p <* 0.001), ∗ ∗ (*p <* 0.01), and ∗ (*p <* 0.05).

We examined spatial immune interactions at 40 *µ*m (as measured by the G-cross function) in Stage III melanoma and their association with survival (**Figure 8**). Several interactions were linked to improved outcomes, suggesting that coordinated adaptive and innate immune responses enhance tumor control (33). These included CTLs attracting M2 macrophages (log-HR = -6.20, *p* = 0.022) and M2 macrophages attracting B cells (log-HR = -2.68, *p* = 0.023), potentially reflecting M1 polarization that promotes anti-tumor immunity (34).

Conversely, interactions linked to worse survival included tumor cells attracting TAMs (log-HR = 1.15, *p* = 0.036), suggesting that macrophage clustering contributes to an immunosuppressive environment. Additionally, CTLs (log-HR = 1.41, *p* = 0.0077), NK cells (log-HR = 1.02, *p* = 0.015), and Th cells (log-HR = 1.72, *p* = 0.0011) attracting tumor cells were associated with poorer outcomes, possibly reflecting ineffective immune surveillance or tumor-driven immune evasion (35).

#### 3.7.2 Spatial interactions in stage IV patients

**Figure 9:**
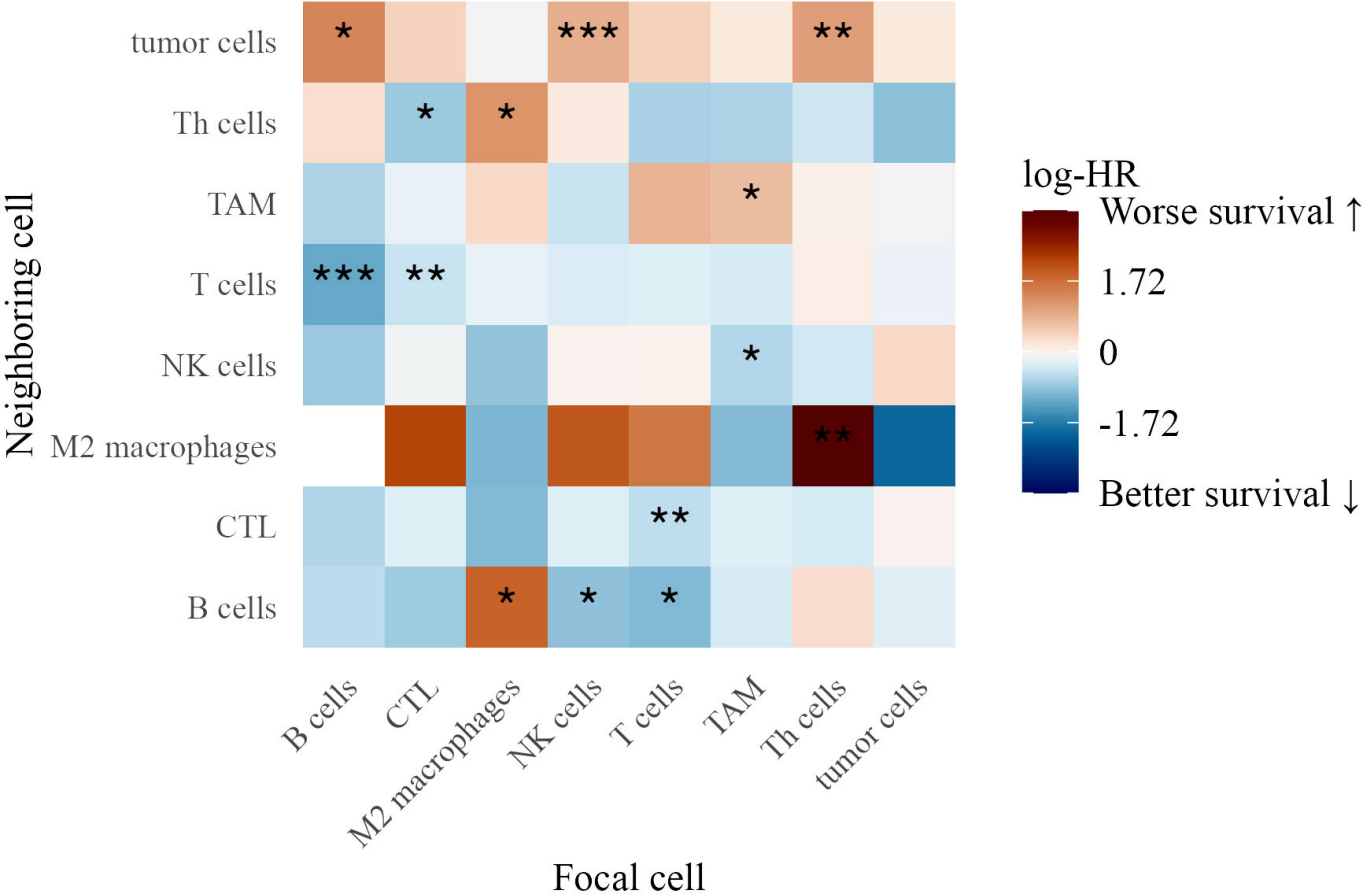
Heatmap of log-hazard ratios estimated using Cox regression, where the G-cross function at 40 *µ*m between each pair of cell types in Stage IV patients serves as a predictor of survival time. Significance levels of hazard ratios are denoted as follows: ∗ ∗ ∗ (*p <* 0.001), ∗ ∗ (*p <* 0.01), and ∗ (*p <* 0.05).

In Stage IV patients, distinct spatial immune interactions correlated with survival (**Figure 9**). Th cells attracting M2 macrophages (log-HR = 3.43, *p* = 0.0029) and tumor cells (log-HR = 1.10, *p* = 0.0034) were associated with worse outcomes, suggesting that Th cells in these contexts contribute to a pro-tumor immune environment.

Conversely, several interactions were linked to better survival, highlighting the role of immune coordination. These included B cells attracting T cells (log-HR = -1.19, *p* = 0.0005), T cells attracting B cells (log-HR = -0.995, *p* = 0.014), and NK cells attracting B cells (log-HR = -0.888, *p* = 0.045), suggesting that reciprocal interactions among adaptive and innate immune populations support anti-tumor immunity. TAMs attracting NK cells (log-HR = -0.616, *p* = 0.020) was also associated with improved survival, potentially indicating that macrophage-NK cell interactions facilitate tumor clearance (36).

T cells attracting CTLs (log-HR = -0.532, *p* = 0.0035) and CTLs attracting T cells (log-HR = -0.473, *p* = 0.0078) reinforced the role of T cell cross-talk in effective tumor control. Additionally, CTLs attracting Th cells (log-HR = -0.814, *p* = 0.044) was linked to improved outcomes, suggesting a coordinated cytotoxic-helper T cell response (37).

The findings from both TMAs underscore the complexity of immune interactions in the tumor microenvironment. While certain immune clusters, such as TAM aggregation and NK cell-tumor cell proximity, were linked to poor survival, interactions among T cells, CTLs, NK cells, and B cells were associated with improved outcomes, highlighting the importance of spatial immune organization in melanoma progression.

### 3.8 Architectural features and cellular neighborhoods associated with inflammatory nodes

We further assessed the spatial clustering and dispersion patterns of immune cell pairs within inflammatory neighborhoods at distances of 20, 40, and 60 *µ*m, using G-cross nearest neighbor estimates and Cox proportional hazards models. These analyses revealed distinct patterns of immune coordination and dysfunction across melanoma progression.

#### 3.8.1 Spatial Inflammatory interactions in stage III patients

**Figure 10:**
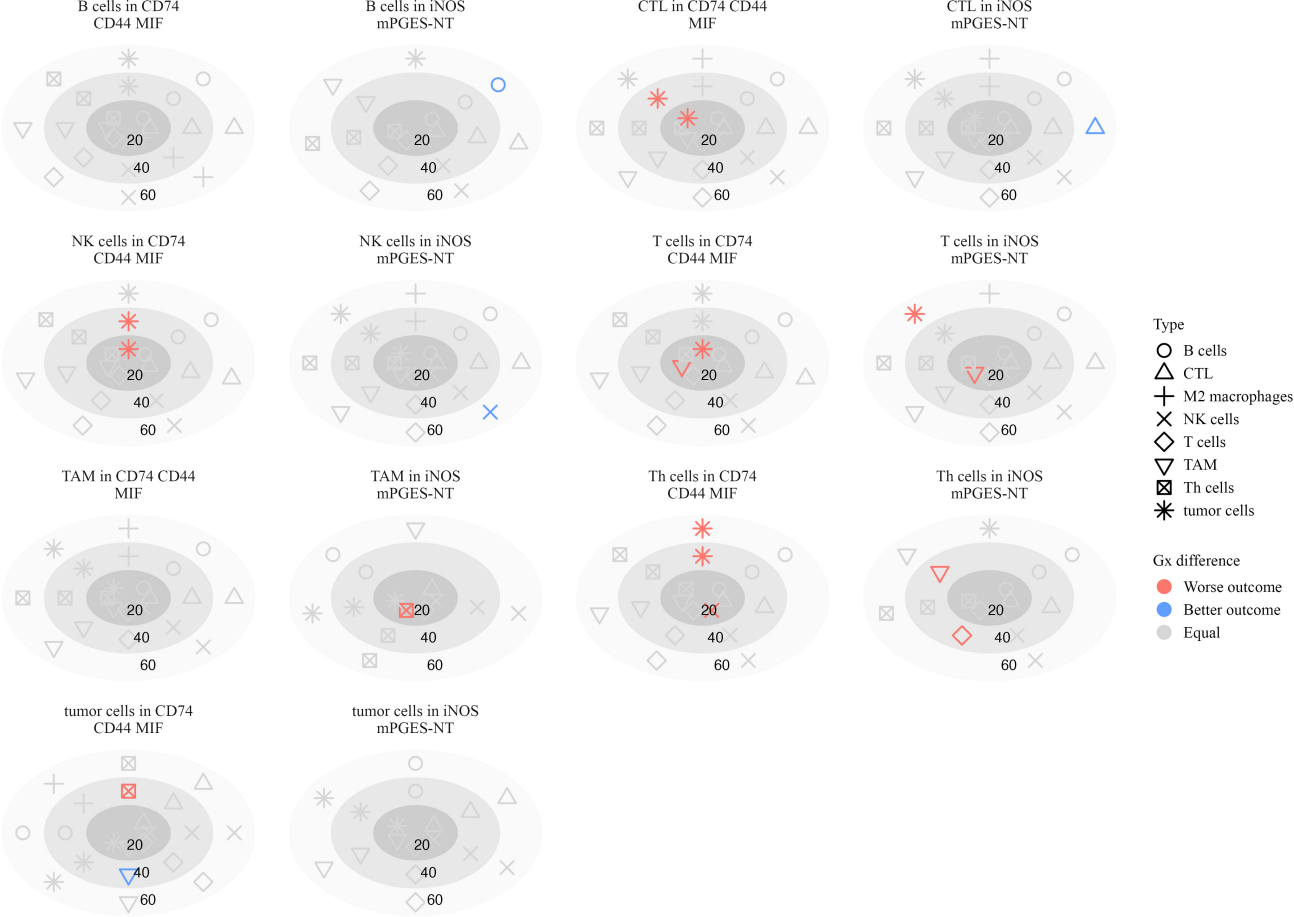
Plots illustrating the significance of hazard ratios (*p <* 0.05) estimated from Cox proportional hazards models, where the G-cross function between each pair of cell types serves as a predictor of survival in stage III patients. Significant associations with worse outcomes are shown in red, while those with better outcomes are shown in blue. Within each subplot, estimates are presented relative to a focal cell type located within a specific inflammatory neighborhood, at radii of 20, 40, and 60 *µ*m.

In Stage III, B cell clustering in iNOS mPGES-NT neighborhoods at 60 *µ*m was associated with better survival (log-HR = -1.69, *p* = 0.016) (**Figure 10**). Similarly, CTLs (log-HR = -1.37, *p* = 0.037) and NK cells (log-HR = -0.867, *p* = 0.027) clustering at 60 *µ*m correlated with improved outcomes, suggesting that cytotoxic immune cells forming structured aggregates enhance anti-tumor responses.

Conversely, interactions associated with poor survival included Th cells attracting T cells (log-HR = 4.73, *p* = 0.0055) and TAMs (log-HR = 2.60, *p <* 0.001) at 40 *µ*m, suggesting that these interactions contribute to an immunosuppressive environment. T cells attracting TAMs at 20 *µ*m (log-HR = 2.78, *p* = 0.0005) were also associated with worse survival, possibly reflecting immune dysfunction or tumor-driven inflammation.

Macrophage interactions in CD74 CD44 MIF neighborhoods exhibited mixed survival associations. Increased clustering of TAMs around T cells at 20 *µ*m (log-HR = 1.94, *p* = 0.0005) was associated with worse survival, consistent with macrophage-mediated suppression of T cell activity (38). Similarly, tumor cells clustering around NK cells at 20 *µ*m (log-HR = 1.10, *p* = 0.0038) and 40 *µ*m (log-HR = 1.11, *p* = 0.022) correlated with worse outcomes, suggesting potential immune evasion mechanisms (39). Th cells clustering with tumor cells at 40 *µ*m (log-HR = 2.22, *p* = 0.0036) and 60 *µ*m (log-HR = 1.78, *p* = 0.013) further supported a role for these interactions in immune escape or chronic inflammation.

#### 3.8.2 Spatial inflammatory interactions in stage IV patients

**Figure 11:**
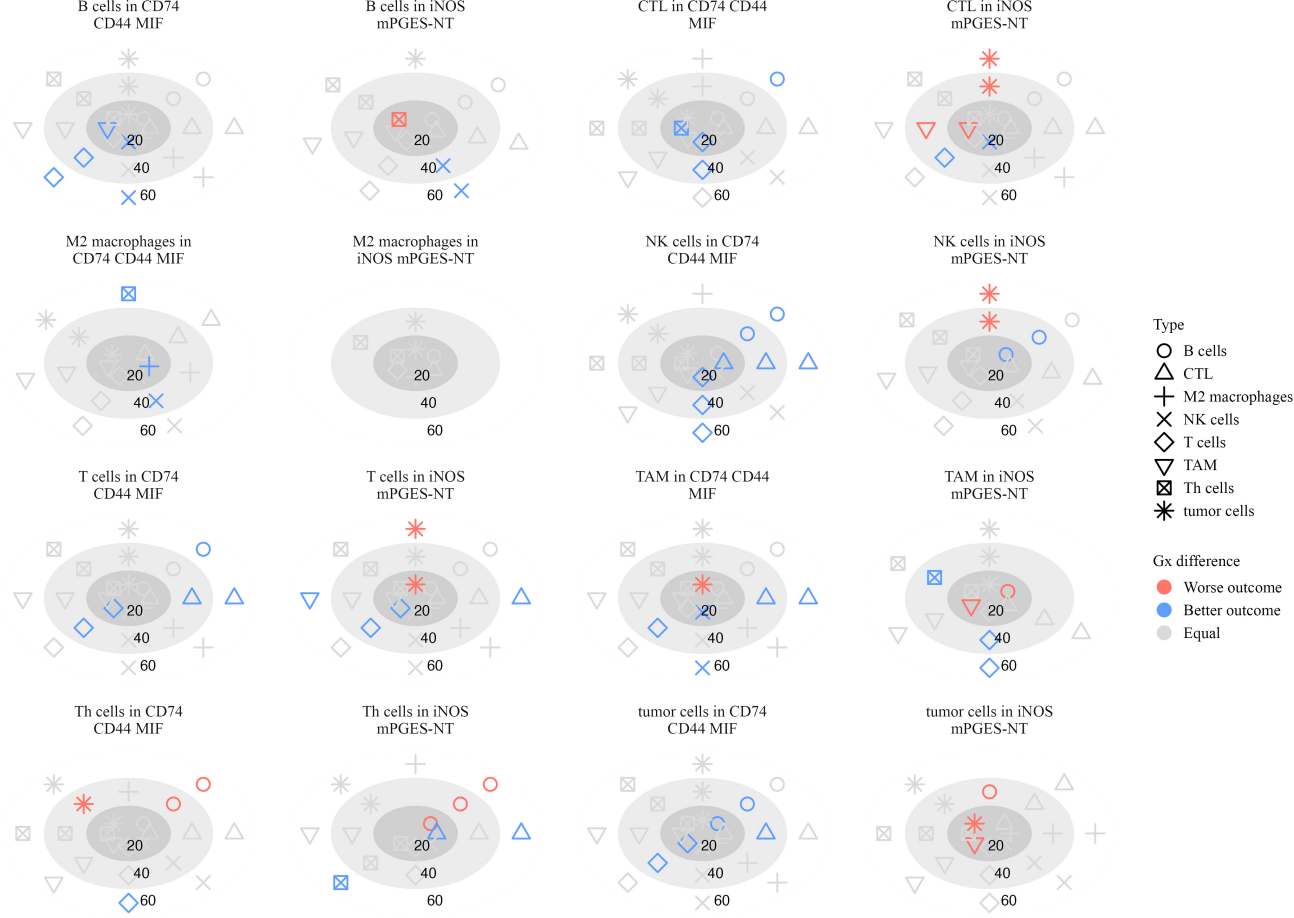
Plots illustrating the significance of hazard ratios (*p <* 0.05) estimated from Cox proportional hazards models, where the G-cross function between each pair of cell types serves as a predictor of survival in stage IV patients. Significant associations with worse outcomes are shown in red, while those with better outcomes are shown in blue. Within each subplot, estimates are presented relative to a focal cell type located within a specific inflammatory neighborhood, at radii of 20, 40, and 60 *µ*m.

In Stage IV melanoma, spatial immune interactions within inflammatory neighborhoods showed significant associations with survival (**Figure 11**). Several interactions were associated with improved outcomes, suggesting that coordinated immune activity plays a role in tumor suppression.

B cells attracting NK cells in CD74 CD44 MIF neighborhoods was linked to better survival at 20 *µ*m (log-HR = -1.48, *p* = 0.033) and 60 *µ*m (log-HR = -1.73, *p* = 0.0018), indicating that NK cell presence close to B cells in these regions may contribute to an effective anti-tumor response. Similarly, NK cells attracting T cells at 40 *µ*m (log-HR = -0.958, *p* = 0.0001) and 60 *µ*m (log-HR = -1.16, *p <* 0.0001) suggests that NK-T cell interactions may play a role in immune surveillance (40, 41).

Additional immune interactions in CD74 CD44 MIF neighborhoods were also linked to better survival. CTLs attracting T cells at 20 *µ*m (log-HR = -0.587, *p* = 0.011) and 40 *µ*m (log-HR = -0.436, *p* = 0.023) suggests that cytotoxic and helper T cell coordination contributes to positive outcomes (37). M2 macrophages attracting NK cells at 40 *µ*m (log-HR = -2.97, *p* = 0.020) and TAMs attracting NK cells at 20 *µ*m (log-HR = -1.18, *p* = 0.050) and 60 *µ*m (log-HR = -0.919, *p* = 0.027) suggests that macrophages in these neighborhoods may recruit NK cells to mediate tumor suppression (42).

In iNOS mPGES-NT neighborhoods, additional immune interactions correlated with improved survival. Th cells attracting Th cells at 60 *µ*m (log-HR = -3.58, *p* = 0.012) and CTLs at 60 *µ*m (log-HR = -3.41, *p* = 0.033) suggests a coordinated immune response rather than immune dysfunction. Additionally, CTLs attracting NK cells at 20 *µ*m (log-HR = -0.939, *p* = 0.024) and B cells attracting NK cells at 60 *µ*m (log-HR = -5.30, *p <* 0.0001) further support the role of cytotoxic cell recruitment in tumor control (36).

Several immune interactions were associated with worse survival, particularly those involving excessive clustering of immune cells within inflammatory neighborhoods. In iNOS mPGES-NT neighborhoods, Th cells attracting B cells at 20 *µ*m (log-HR = 7.53, *p* = 0.028) and 40 *µ*m (log-HR = 2.04, *p* = 0.0007) was linked to poor prognosis, suggesting that excessive B cell clustering around Th cells in these regions may contribute to immune dysregulation rather than an effective anti-tumor response. Similarly, TAMs attracting B cells at 20 *µ*m (log-HR = 5.71, *p* = 0.048) and tumor cells attracting TAMs at 20 *µ*m (log-HR = 4.03, *p* = 0.0006) were associated with poor survival, reinforcing the idea that macrophages in these settings may promote tumor progression rather than restrict it (43, 44).

Th cells had several interactions in CD74 CD44 MIF neighborhoods that were associated with poor patient outcomes. Specifically, Th cells attracting B cells at 60 *µ*m (log-HR = 1.39, *p* = 0.03) suggests that B cell clustering around Th cells in this neighborhood may not contribute to an effective anti-tumor response. Similarly, Th cells attracting tumor cells at 40 *µ*m (log-HR = 0.973, *p* = 0.023) indicates that these interactions may facilitate immune evasion or chronic inflammation.

These findings underscore the role of spatial immune organization in shaping patient outcomes. While interactions involving NK cells, CTLs, and macrophages were linked to improved survival, excessive B cell and T cell clustering within pro-inflammatory neighborhoods, as well as macrophage recruitment, were associated with immune dysfunction and worse prognosis in advanced melanoma.

### 3.9 Spatial immune infiltration across metastatic sites and its impact on patient survival

In our final analysis, we examined how the spatial infiltration patterns of immune and tumor cells within different subsets of melanoma metastases influence patient survival. We analyzed infiltration behavior in five metastatic sites: lung, gastrointestinal tract (GI), skin, lymph nodes, and a combined “other” category, which included organs with insufficient sample sizes for individual analysis. Using survival models at infiltration radii of 20, 40, 60, and 80 *µ*m, we distinguished between nearest-cell-neighbor interactions at smaller distances and broader immune neighborhood effects at larger scales.

#### 3.9.1 GI metastases: CTL-tumor interactions predict survival

In GI metastases (**Supplementary Figure S2**), most significant survival correlations exhibited a positive trend, indicating that greater immune cell infiltration around focal cell populations generally corresponded to improved patient outcomes. Notably, increased CTL-CTL clustering was strongly associated with better survival across multiple distances (*p*(20) = 0.0179, *p*(40) *<* 0.001, *p*(60) = 0.0048, *p*(80) *<* 0.001). Similarly, higher tumor-CTL infiltration at 20 *µ*m (*p <* 0.001) was linked to improved outcomes, suggesting that direct tumor engagement by cytotoxic T cells enhances anti-tumor responses (45–47). However, when the focal cell type was reversed (i.e., tumor cells clustering near T cells instead of T cells clustering near tumor cells), this interaction at 20 *µ*m was significantly associated with worse survival (*p* = 0.0309). This suggests that tumor-driven immune evasion mechanisms, such as immune checkpoint upregulation or T cell exhaustion, may be at play in poor prognosis patients.

#### 3.9.2 Lung, skin, and lymph node metastases: NK and T cell interactions favor survival

For lung, skin, and lymph node (LN) metastases (**Supplementary Figures S3, S4, S5**, respectively), most hazard ratio trends indicated that increased immune cell infiltration correlated with better survival. A key finding in LN metastases was the increased infiltration of NK cells around T cells, which was significantly linked to improved survival across all distances (*p*(20) *<* 0.001, *p*(40) *<* 0.001, *p*(60) *<* 0.001, *p*(80) *<* 0.001). This may reflect an enhanced immunosurveillance role of NK cells in the lymph node microenvironment, where they can activate T cells and promote cytotoxic responses (48, 49). Additionally, greater tumor cell infiltration near CTLs at larger distances (40–80 *µ*m) was associated with improved survival (*p*(40) *<* 0.001, *p*(60) *<* 0.001, *p*(80) *<* 0.001), suggesting that CTL-mediated tumor clearance may still be effective in these microenvironments (50).

#### 3.9.3 Other metastatic sites: tumor clustering and immune surveillance

In metastases from other sites (**Supplementary Figure S6**), increased infiltration of several immune-tumor cell pairs correlated with better survival. This included tumor-T cell interactions (*p*(40) = 0.0093, *p*(60) = 0.0305), NK cell-CTL clustering (*p*(20) = 0.0153, *p*(40) *<* 0.001, *p*(60) = 0.0317, *p*(80) = 0.0356), and B cell-tumor interactions at larger distances (*p*(60) *<* 0.001, *p*(80) *<* 0.001). However, increased tumor- tumor clustering at higher distances was associated with worse outcomes (*p*(40) = 0.046, *p*(60) = 0.0305, *p*(80) = 0.0308), potentially reflecting an immunosuppressive niche that limits immune cell infiltration (51).

## 4 Discussion

The tumor immune microenvironment (TIME) plays a crucial role in melanoma progression and treatment response. Understanding the spatial organization of immune cells within inflammatory neighborhoods can reveal mechanisms of immune suppression, evasion, and activation that influence patient outcomes. This study provides a comprehensive analysis of immune spatial architecture in metastatic melanoma, highlighting key differences between Stage III and Stage IV disease and identifying immune interactions linked to survival.

### 4.1 Spatial immune reorganization in stage IV melanoma

Our results indicate a significant reorganization of immune cell distributions in Stage IV melanoma compared to Stage III. CD74- and MIF-enriched inflammatory neighborhoods were more prevalent in Stage IV, while iNOS-associated neighborhoods were significantly reduced. These findings suggest a shift from oxidative stress-driven inflammation in Stage III toward CD74-MIF-associated immune modulation in advanced disease. Previous studies have implicated CD74-MIF signaling in promoting tumor progression by suppressing cytotoxic immune responses and enhancing macrophage-mediated immune evasion (30, 31).

Stage IV tumors exhibited a depletion of B cells and NK cells across multiple inflammatory neighborhoods, while cytotoxic T lymphocytes (CTLs) were more frequently found in CD74-MIF regions. This suggests that despite increased CTL presence, their effectiveness may be diminished in an environment enriched for immune-modulatory signals (16). Tumor cells were also significantly more localized within CD74 MIF and MIF CD44 neighborhoods, reinforcing the role of these regions as hubs of immune suppression.

### 4.2 Immune interactions and survival outcomes

We identified distinct immune interactions that correlated with patient survival. In Stage III melanoma, interactions involving B cells, macrophages, and CTLs were linked to improved outcomes. Specifically, CTLs attracting M2 macrophages and M2 macrophages attracting B cells were associated with better survival, suggesting that coordinated innate and adaptive immune responses contribute to tumor control (33, 34). Conversely, interactions where tumor cells attracted TAMs, and macrophages clustered within tumors, were associated with worse prognosis, supporting prior evidence that macrophage accumulation can promote immune evasion and tumor progression (35).

In Stage IV melanoma, cytotoxic immune interactions remained important for survival. B cells attracting NK cells and NK cells attracting T cells were associated with improved outcomes, suggesting that NK cells play a role in sustaining immune surveillance. Similarly, CTLs attracting Th cells were linked to better survival, reinforcing the importance of coordinated adaptive immune responses (37). These findings align with recent work demonstrating that effective tumor control relies on dynamic crosstalk between cytotoxic and helper T cell populations.

Conversely, several immune interactions were associated with worse survival, particularly those involving excessive clustering of immune cells within inflammatory neighborhoods. In iNOS mPGES-NT neighborhoods, Th cells attracting B cells at short distances correlated with poor prognosis, suggesting that excessive B cell clustering in these regions may contribute to immune dysfunction rather than anti-tumor immunity. Similarly, TAMs attracting B cells and tumor cells attracting TAMs were linked to worse survival, reinforcing the idea that macrophages in these contexts may promote tumor progression rather than restrict it (43, 44).

### 4.3 TAM and CTL dynamics in the TIME

Our spatial analysis of G-cross nearest neighbor interactions provided additional insights into the complex roles of macrophages and CTLs in melanoma. While some TAM-CTL interactions were linked to better survival, excessive TAM accumulation was associated with poorer outcomes. This asymmetry suggests that TAMs can either support or suppress anti-tumor responses depending on their activation state and spatial organization within the tumor bed. Previous work has shown that TAMs can adopt both pro- and anti-inflammatory phenotypes depending on local signaling cues (42). Our findings further emphasize the need to functionally characterize macrophage subsets in melanoma tumors to determine their precise role in immune regulation.

Another key observation was the differential immune cell composition between Stage III and Stage IV tumors. Stage III tumors exhibited a higher proportion of naive T cells, helper T cells, NK cells, and B cells, whereas Stage IV tumors were dominated by CTLs and TAMs. While increased CTL infiltration is often associated with improved prognosis, our data suggest that not all CTLs in Stage IV tumors exhibit effective cytotoxic function. Emerging studies indicate that tumor-infiltrating CTLs can become dysfunctional or exhausted in immunosuppressive microenvironments, reducing their efficacy (47). The prolonged interactions observed between CTLs and TAMs in Stage IV may further contribute to T cell dysfunction.

### 4.4 Clinical and therapeutic implications

These findings have important implications for immunotherapy strategies. Given the enrichment of CTLs in CD74- and MIF-associated neighborhoods in Stage IV tumors, interventions targeting these pathways may help restore CTL function and improve therapeutic outcomes. Previous studies have shown that blocking CD74-MIF signaling can enhance T cell infiltration and reduce tumor growth (30). Similarly, targeting mPGES1, which we identified as a key player in immune evasion of melanoma, may enhance responses to immune checkpoint inhibitors (12).

The spatial organization of immune cells may also inform patient stratification for immunotherapy. Our results suggest that patients with increased NK cell-B cell interactions or structured CTL-Th cell clustering may have better responses to immune-based treatments. Conversely, patients with excessive macrophage clustering or high B cell accumulation in inflammatory regions may require combination therapies that target these immunosuppressive niches.

### 4.5 Study strengths and limitations

The strengths of this study lie in the size of our cohort and the high-resolution spatial analysis of immune interactions. By integrating multiplex imaging with computational modeling, we were able to identify key differences in immune cell organization between Stage III and IV melanoma. However, several limitations should be noted. First, the retrospective nature of this study introduces potential biases related to treatment variability. Due to the specifics of these TMA cohorts’ establishment, we were limited in the clinical information available, and, as a result, our conclusions carefully refrain from making any statements on treatment outcomes or responses to therapy. Therefore, our focus remains on the differences in TIME immunobiology between stages, which may inform future decisions regarding IO approaches in clinical trials. Additionally, while our analyses reveal associations between spatial immune patterns and survival, they do not establish causal relationships. Further functional validation is required to determine how specific immune interactions influence tumor progression and response to therapy.

### 4.6 Conclusions and future directions

Our findings underscore the importance of spatial immune organization in melanoma progression and treatment response. We identified distinct immune interactions that correlate with survival, highlighting the roles of cytotoxic immune coordination and macrophage-driven immune suppression in shaping patient outcomes. Future studies should focus on validating these findings in independent cohorts and exploring therapeutic strategies that modulate immune cell spatial organization to improve clinical responses.

## Data Availability

The original contributions presented in the study are included in the article/**Supplementary Material**. Further inquiries can be directed to the corresponding author.
