# Supplementary material for: Characterizing spatial immune architecture in metastatic melanoma using high-dimensional multiplex imaging
Source: Front Immunol. 2025 Apr 29;16:1560778. doi: 10.3389/fimmu.2025.1560778 (PMC12069457; doi:10.3389/fimmu.2025.1560778)
Supplement: Supplementary file 1 [file DataSheet1.pdf]

frontiers

Supplementary Material

1 SUPPLEMENTARY TABLES AND FIGURES

1.1 Figures

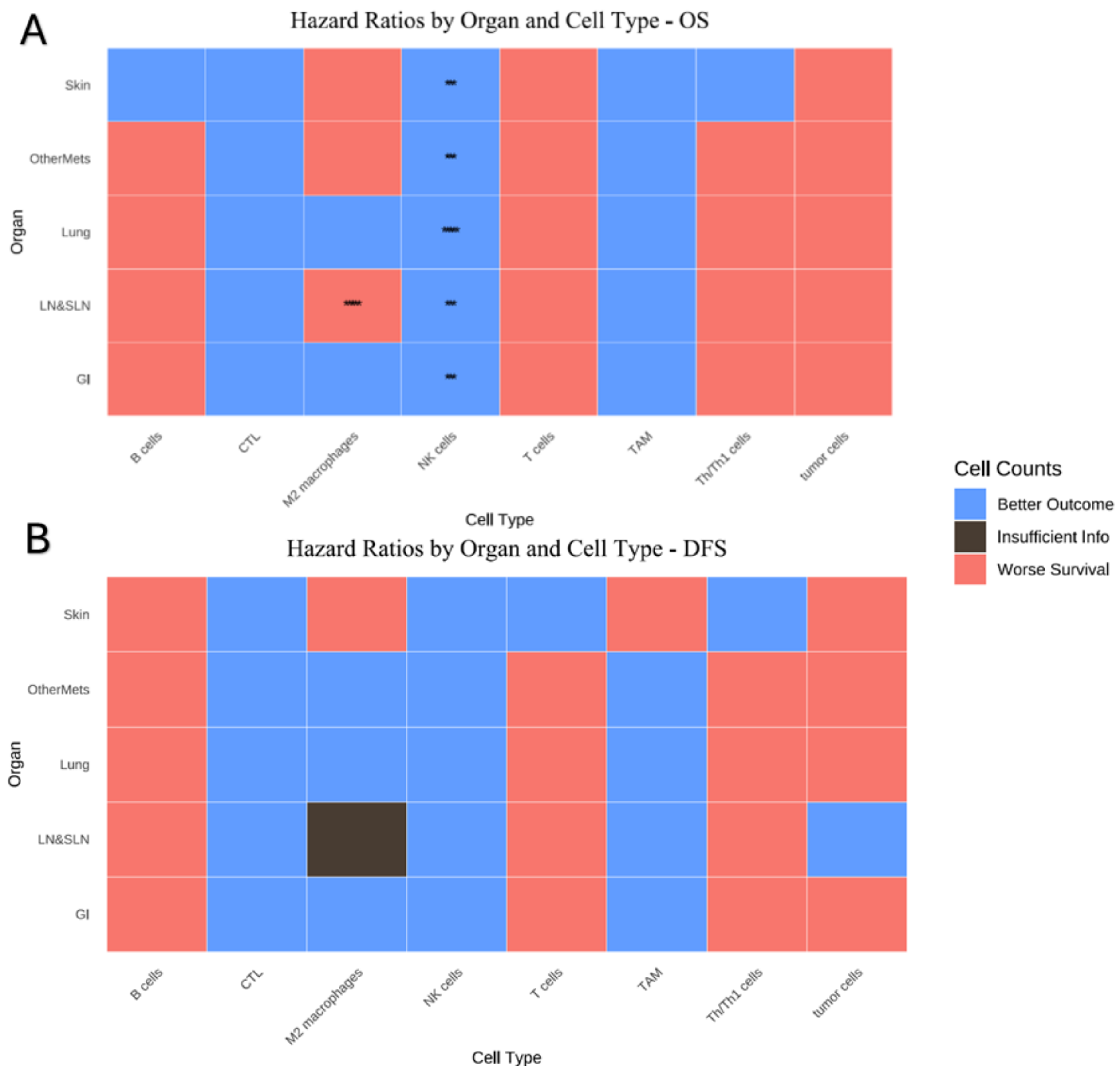

Figure S1: Heatmaps of hazard ratios estimated using Cox regression, where the raw cell counts of each cell type serves as a predictor of (A) Overall Survival(OS) and (B) Disease-Free Survival in 5 different organ metastases, as determined in our study. Significance levels of hazard ratios are denoted as follows: $***(p < 0.001)$, $**\ (p < 0.01)$, and $*(p < 0.05)$.

We next computed log-hazard ratios

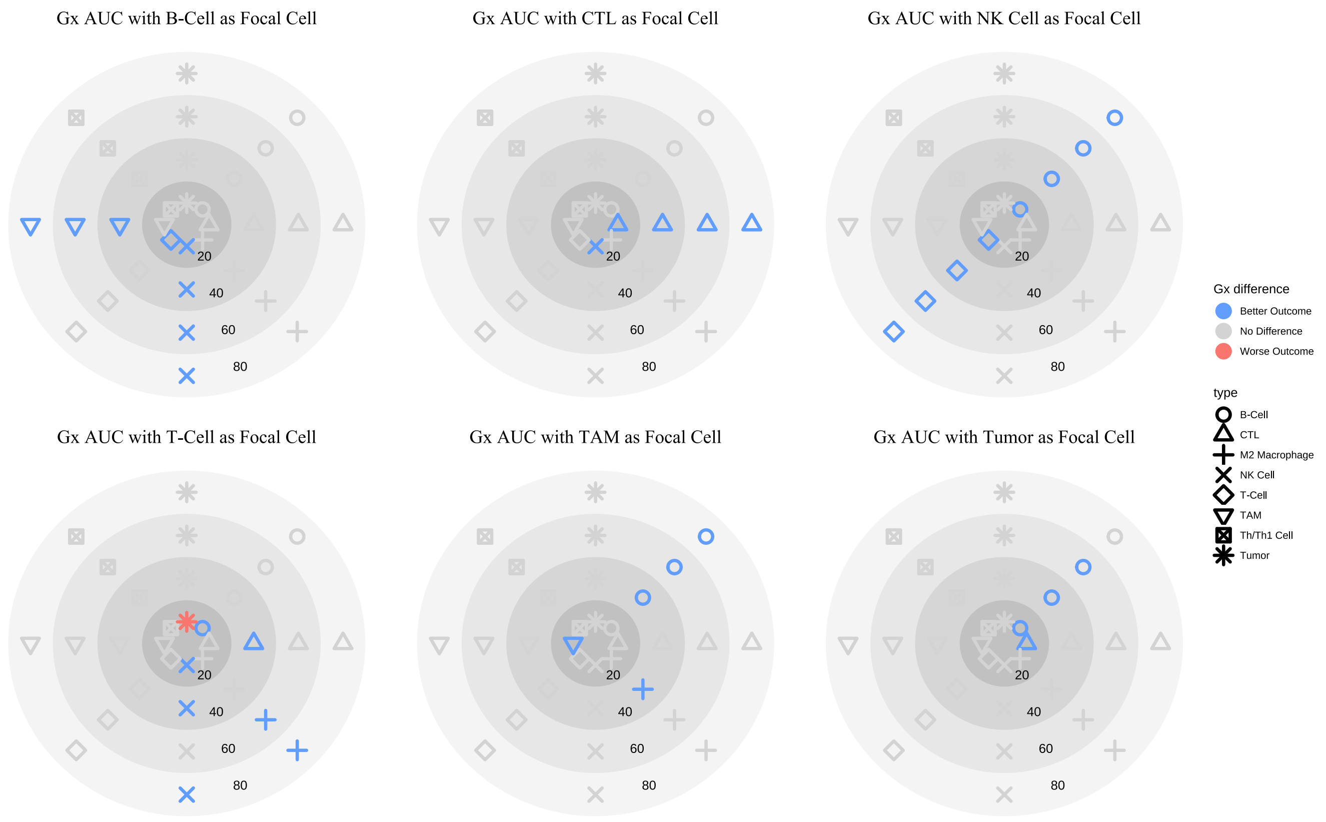

Figure S2: Cell-type specific plots showing whether hazard ratios, as estimated from Cox proportional hazards model(with computed Gcross AUCs as predictors in a survival model) are significant in Stage 4 patient GI metastases, and if so, whether they are associated with worse(red) or better(blue) patient outcomes. The AUC values are computed at radii of 20, 40, 60 and 80 μm. p-values are NOT FDR-adjusted.

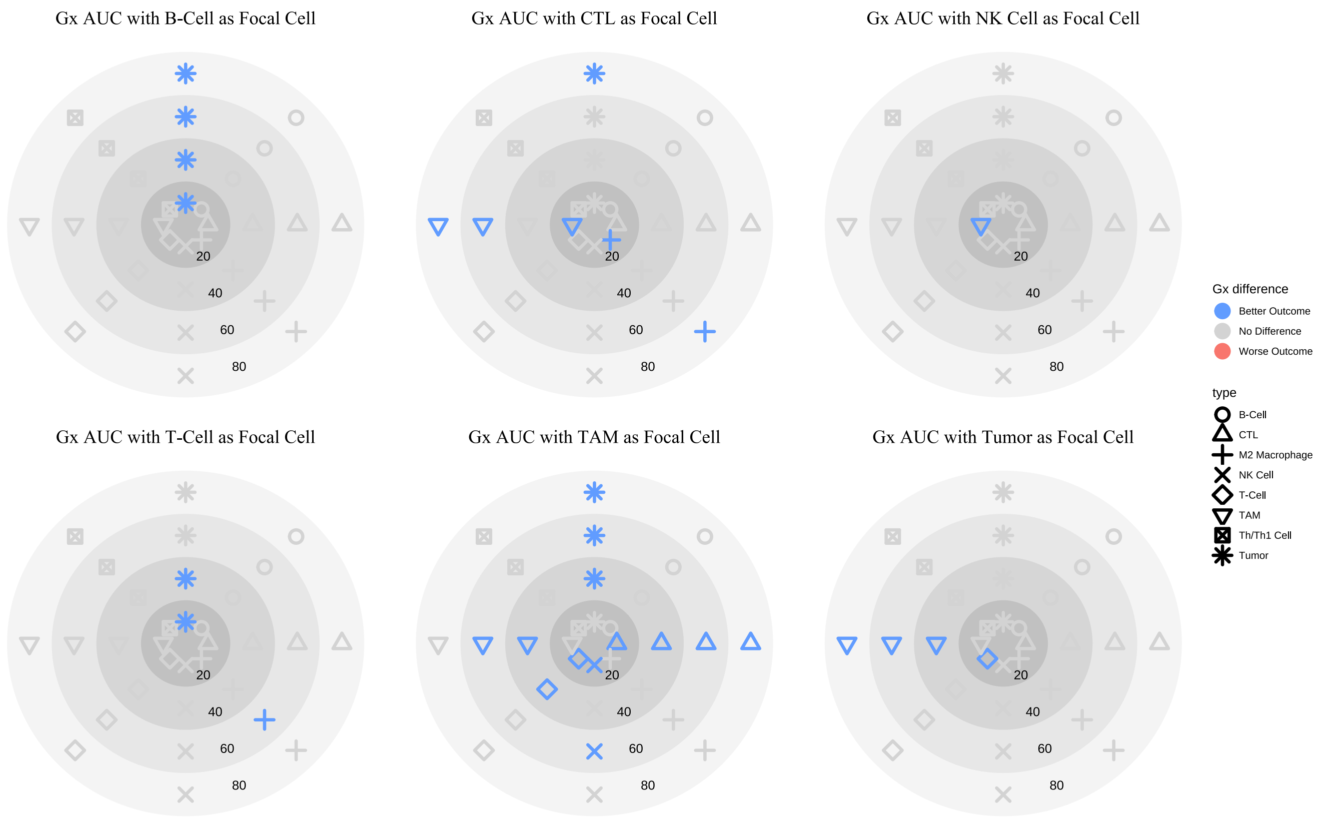

Figure S3: Cell-type specific plots showing whether hazard ratios, as estimated from Cox proportional hazards model(with computed Gcross AUCs as predictors in a survival model) are significant in Stage 4 patient Lung metastases, and if so, whether they are associated with worse(red) or better(blue) patient outcomes. The AUC values are computed at radii of 20, 40, 60 and 80 μm. p-values are NOT FDR-adjusted.

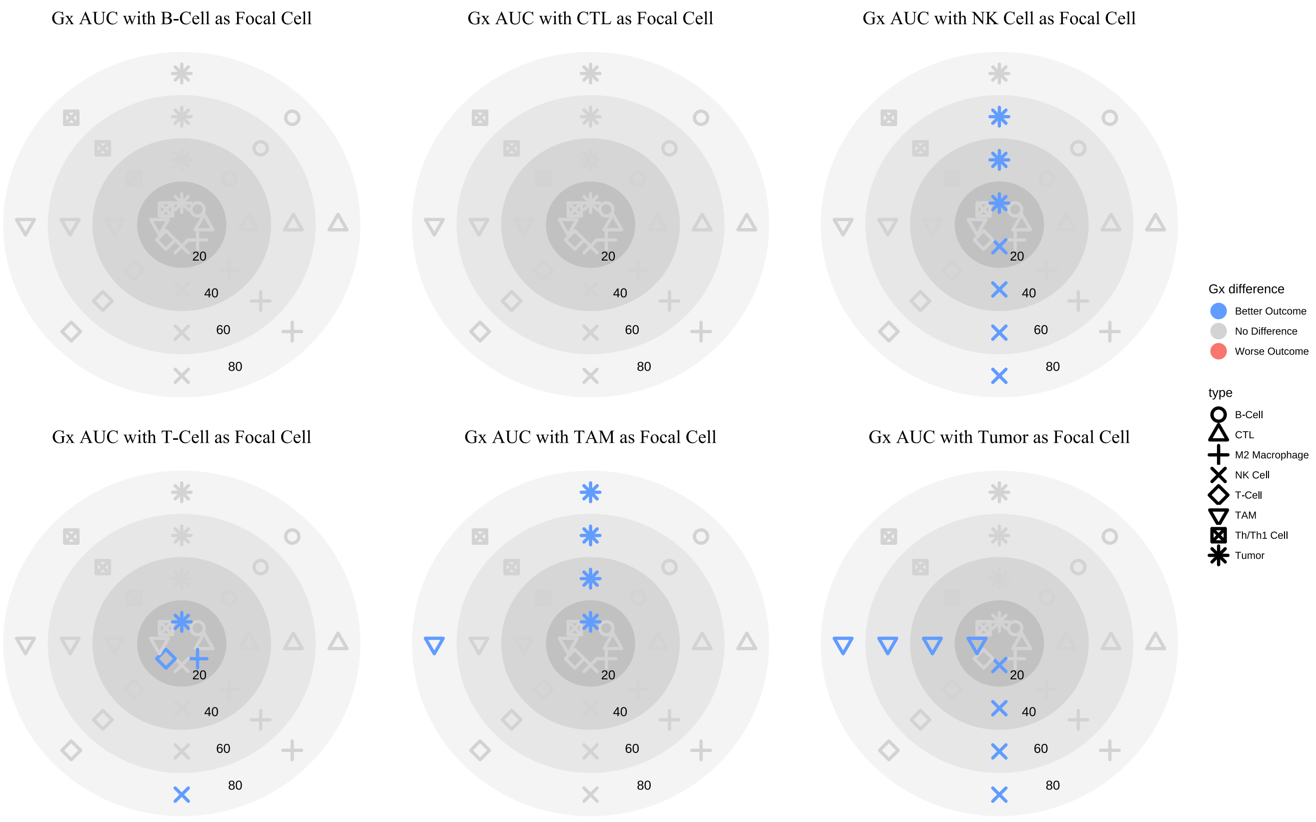

Figure S4: Cell-type specific plots showing whether hazard ratios, as estimated from Cox proportional hazards model(with computed Gcross AUCs as predictors in a survival model) are significant in Stage 4 patient Skin metastases, and if so, whether they are associated with worse(red) or better(blue) patient outcomes. The AUC values are computed at radii of 20, 40, 60 and 80 μm. p-values are NOT FDR-adjusted.

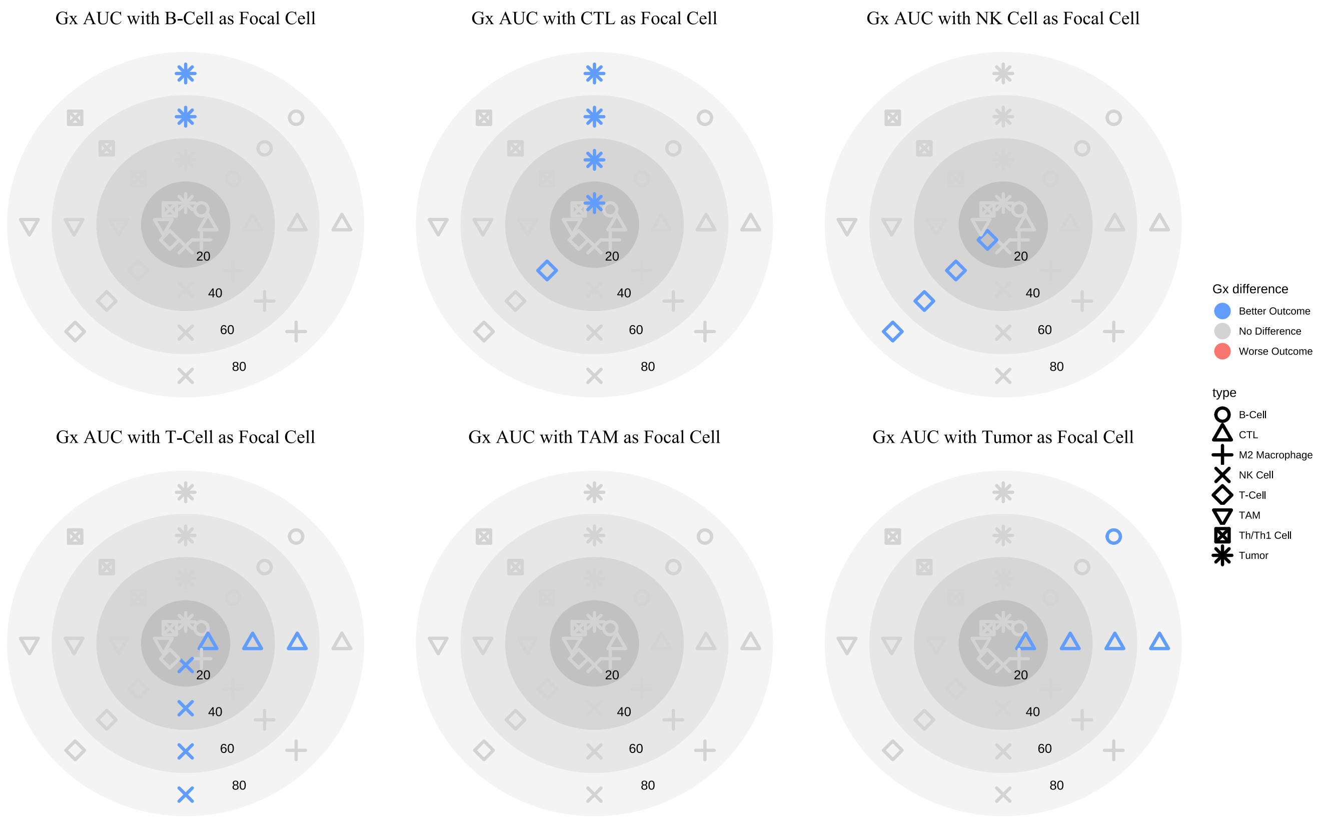

Figure S5: Cell-type specific plots showing whether hazard ratios, as estimated from Cox proportional hazards model(with computed Gcross AUCs as predictors in a survival model) are significant in Stage 4 patient Lymph Node metastases, and if so, whether they are associated with worse(red) or better(blue) patient outcomes. The AUC values are computed at radii of 20, 40, 60 and 80 μm. p-values are NOT FDR-adjusted.

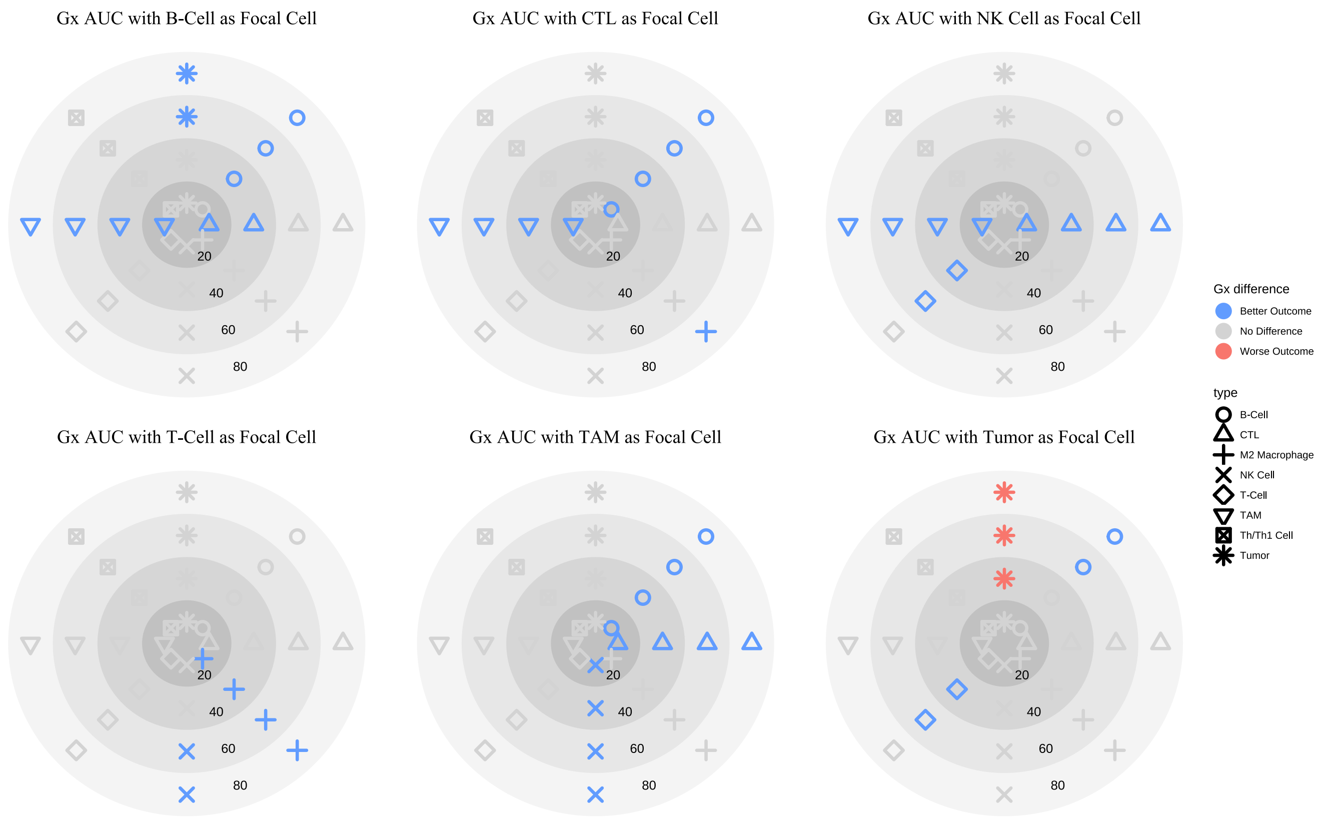

Figure S6: Cell-type specific plots showing whether hazard ratios, as estimated from Cox proportional hazards model(with computed Gcross AUCs as predictors in a survival model) are significant in Stage 4 patient metastases at other locations, and if so, whether they are associated with worse(red) or better(blue) patient outcomes. The AUC values are computed at radii of 20, 40, 60 and 80 μm. p-values are NOT FDR-adjusted.

1.2 Tables

Table S1. List of abbreviations used in this study.

Abbreviation	Definition
ICI	Immune Checkpoint Inhibitors
TIME	Tumor Immune Microenvironment
TMA	Tissue Microarray
FFPE	Formalin-Fixed Paraffin-Embedded
H & E	Hematoxylin and Eosin
CyTOF	Cytometry by Time-of-Flight
IMC	Imaging Mass Cytometry
t-SNE	t-distributed Stochastic Neighbor Embedding
mIF	Multiplex Immune Fluorescence
MCD	miniCAD Design
TAM	Tumor-Associated Macrophages
CTL	Cytotoxic T Cells
NK	Natural Killer
MIF	Macrophage Inhibitory Factors
iNOS	Inducible Nitric Oxide Synthase
NT	Nitrotyrosine
mPGES	Microsomal Prostaglandin E Synthase-1
COX-2	Cyclooxygenase-2
PGE2	Prostaglandin E2

Table S2. List of the 35 antibodies labeled with unique metal isotopes

Select	Mass	Targets	Clone	Source
1	^{139}La	CXCR2	5E8	BioLegend
2	^{141}Pr	PTEN	4C11A11	BioLegend
3	^{142}Nd	CD19	6OMP31	DVS-Fluidigm
4	^{143}Nd	Vimentin	RV202	DVS-Fluidigm
5	^{144}Nd	p-Tyr	p-Tyr-100	DVS-Fluidigm
6	^{145}Nd	T-bet	D6N8B	DVS-Fluidigm
7	^{146}Nd	CD8a	RPA-T8	BioLegend
8	^{147}Sm	HIF-1a	BL-124-3F7	BETHYL
9	^{148}Nd	OX40	Ber-ACT35	BioLegend
10	^{149}Sm	CD11b	EPR1344	DVS-Fluidigm
11	^{150}Nd	PD-L1	E1L3N	DVS-Fluidigm
12	^{151}Eu	CD31	EPR3094	DVS-Fluidigm
13	^{152}Sm	CD45	D9M8I	DVS-Fluidigm
14	^{153}Eu	SOX10	BLR080G	BETHYL
15	^{154}Sm	CD11c	Polyclonal	DVS-Fluidigm
16	^{155}Gd	FoxP3	BLR034F	BETHYL
17	^{156}Gd	CD4	EPR6855	DVS-Fluidigm
18	^{159}Tb	CD68	KP1	DVS-Fluidigm
19	^{160}Gd	CD44	IM7	BioLegend
20	^{161}Dy	CD20	Polyclonal	DVS-Fluidigm
21	^{162}Dy	CD8a	D8A8Y	DVS-Fluidigm
22	^{163}Dy	VEGF	G153-694	DVS-Fluidigm
23	^{164}Dy	ARG1	BLR036F	BETHYL
24	^{165}Ho	PD-1	EPR4877(2)	DVS-Fluidigm
25	^{166}Er	HLA-ABC	W6/32	BioLegend
26	^{167}Er	Granzyme B	EPR20129-217	DVS-Fluidigm
27	^{168}Er	Ki67	BLR021E	BETHYL
28	^{169}Tm	CXCR4	12G5	BioLegend
29	^{170}Er	CD3	Polyclonal	DVS-Fluidigm
30	^{171}Yb	p-ERK1/2	D13.14.4E	DVS-Fluidigm
31	^{172}Yb	PD-L2	D7U8C	DVS-Fluidigm
32	^{173}Yb	EOMES	644730	R&D
33	^{174}Yb	HLA-DR	L243	BioLegend
34	^{175}Lu	S100A9	Polyclonal	Proteintech
35	^{176}Yb	CD56	NCAM16.2	BD

Table S3. Survival statistics for stage III TMA patients

Variable	Value
**Median Overall Survival (months)**	20.2 (95% CI: 15.3–61.7)
**5-Year Survival Probability**	41.5%
**10-Year Survival Probability**	27.9%
**Censoring Rate**	26.5%

Table S4. Overall survival statistics for stage IV TMA patients

Variable	Value
**Median Overall Survival (months)**	10.2 (95% CI: 8.6–12.4)
**5-Year Survival Probability**	6.8%
**10-Year Survival Probability**	2.6%
**Censoring Rate**	6.0%

Table S5. Disease-free survival statistics for stage IV TMA patients

Variable	Value
**Median Disease-Free Survival (months)**	18.7 (95% CI: 15.0–29.2)
**5-Year Survival Probability**	31.8%
**10-Year Survival Probability**	20.2%
**Censoring Rate**	62.5%

Cell Phenotypes	Cellular Markers Included
Proliferating cells (Any)	$Ki67^+$
Tumor Cells	$SOX10^+$ and/or $S100A9^+$
Lymphocytes	$CD45^+$
Myeloid Cells	$CD45^+$ $CD11b^+$
Macrophages	$CD45^+$ $CD11b^+$ $CD11c^-$ $CD68^+$
M2 macrophages	$CD45^+$ $CD11b^+$ $CD11c^-$ $CD68^+$ $Arg1^+$
B cells	$CD45^+$ $CD19^+$ and/or $CD20^+$
T cells	$CD45^+$ $CD3^+$
$CD4^+$ T cells	$CD45^+$ $CD3^+$ $CD4^+$
$CD8^+$ T cells	$CD45^+$ $CD3^+$ $CD8^+$ and/or $GRZB^+$
NK cells	$CD45^+$ $CD3^-$ $CD56^+$
Th1 cells	$CD45^+$ $CD3^+$ $CD4^+$ $Tbet^+$

Table S6. Cell phenotypes and their associated cellular markers.
